# Changes in patterns of mortality rates and years of life lost due to firearms in the United States, 1999 to 2016: A joinpoint analysis

**DOI:** 10.1371/journal.pone.0225223

**Published:** 2019-11-22

**Authors:** Hannah M. Bailey, Yi Zuo, Feng Li, Jae Min, Krishna Vaddiparti, Mattia Prosperi, Jeffrey Fagan, Sandro Galea, Bindu Kalesan

**Affiliations:** 1 Department of Medicine, Boston University School of Medicine, Boston, Massachusetts, United States of America; 2 School of Statistics and Mathematics, Central University of Finance and Economics, Beijing, China; 3 Department of Epidemiology, College of Public Health & Health Professions, University of Florida, Gainesville, Florida, United States of America; 4 Department of Epidemiology, Mailman School of Public Health and Columbia Law School, New York, New York, United States of America; 5 Department of Epidemiology, Boston University School of Public Health, Boston, Massachusetts, United States of America; Florida State University College of Medicine, UNITED STATES

## Abstract

**Background:**

Firearm-related death rates and years of potential life lost (YPLL) vary widely between population subgroups and states. However, changes or inflections in temporal trends within subgroups and states are not fully documented. We assessed temporal patterns and inflections in the rates of firearm deaths and %YPLL due to firearms for overall and by sex, age, race/ethnicity, intent, and states in the United States between 1999 and 2016.

**Methods:**

We extracted age-adjusted firearm mortality and YPLL rates per 100,000, and %YPLL from 1999 to 2016 by using the WONDER (Wide-ranging Online Data for Epidemiologic Research) database. We used Joinpoint Regression to assess temporal trends, the inflection points, and annual percentage change (APC) from 1999 to 2016.

**Results:**

National firearm mortality rates were 10.3 and 11.8 per 100,000 in 1999 and 2016, with two distinct segments; a plateau until 2014 followed by an increase of APC = 7.2% (95% CI 3.1, 11.4). YPLL rates were from 304.7 and 338.2 in 1999 and 2016 with a steady APC increase in %YPLL of 0.65% (95% CI 0.43, 0.87) from 1999 to an inflection point in 2014, followed by a larger APC in %YPLL of 5.1% (95% CI 0.1, 10.4). The upward trend in firearm mortality and YPLL rates starting in 2014 was observed in subgroups of male, non-Hispanic blacks, Hispanic whites and for firearm assaults. The inflection points for firearm mortality and YPLL rates also varied across states.

**Conclusions:**

Within the United States, firearm mortality rates and YPLL remained constant between 1999 and 2014 and has been increasing subsequently. There was, however, an increase in firearm mortality rates in several subgroups and individual states earlier than 2014.

## Introduction

The burden of death due to firearm violence is a significant public health problem in the United States.[1] While national trends in firearm deaths have remained steady from 1999 to 2014 at around 10.2 per 100,000 persons,[1–3] data from 2015 onwards show increases in the numbers and rates of both fatal and non-fatal firearm-related injuries.[2] There is also a high degree of heterogeneity in firearm mortality among different population groups such as male, white race for firearm suicides, and among blacks for firearm homicide and between individual states.[3] In terms of population groups, higher rates of firearm mortality are seen among men, when compared to women, and among young adults, when compared to older adults.[3] Differences in firearm mortality between states and the intent of injury are also pronounced.[3] Due to the pandemic nature of firearm injuries, the scientific community has called for improved public health surveillance and research by rigorous, objective scientists within governmental public health or academic institutions.[4]

Years of potential life lost (YPLL) before 65 years of age is an estimate of the potential years a person would have lived if they die before the age of 65 years. The changes in YPLL due to premature firearm deaths across the years has not been determined in the U.S. population. Moreover, whether there are changes or inflections in temporal trends in the overall population and subgroups of sex, race/ethnicity, age, and the intent of firearm mortality and YPLL is also unknown. Given the lack of comprehensive comparison between firearm death rates and YPLL in different sub-groups, we performed a hypothesis generating study and assessed age-adjusted firearm mortality rates and rates of YPLL before age 65 to determine temporal trends from 1999 to 2016 and whether there are any inflections in the temporal patterns. We analyzed these changes among subgroups classified by sex, age, race/ethnicity, and intent of injury. Additionally, we assessed temporal trends and inflections in temporal trends in firearm mortality and firearm YPLL rates in all fifty U.S. states and the District of Columbia from 1999 to 2016.

## Materials and methods

Boston University Ethics Board approved the study (H-36195).

### Data source

The Web-based Injury Statistics Query and Reporting System (WISQARS) was used to extract overall, sex-specific, age group-specific, race/ethnicity-specific, intent-specific, and state-specific age-adjusted firearm-related fatal injuries rates,[2] as well as YPLL rates per 100,000 and %YPLL [2] annually from 1999 to 2016. Boston University Ethics Board approved the study (H-36195).

### Study design

Repeated cross-sectional study design was used to assess the national, state-specific, and population subgroup-specific temporal patterns in firearm mortality and YPLL rates, and %YPLL from 1999 as the baseline year to 2016. All US states provided Hispanic origin mortality data at sufficient levels starting in 1997.[2] Additionally, International Classification of Disease (ICD) version 9 was used from 1981 to 1998 to define intent of injury. ICD-10 was used to identify intent of injury starting from 1999.[2] Therefore, we used data from 1999 due to availability of Hispanic data and use of ICD-10 for uniformity in data collection.

### Variables

Age-adjusted firearm-related fatal injury, YPLL rates, and % of firearm YPLL in all-cause mortality were exported from WISQARS from 1999 to 2016. ICD-10 codes used for identifying firearm deaths were W32, W33, W34 (unintentional), X72, X73, X74 (suicide), X93, X94, X95 (homicide/assault), Y22, Y23, Y24 (undetermined), and Y35.0 (legal intervention). WISQARS calculates age-adjusted rates by the direct method and standardizes them to the total U.S. population during the year 2000 using 5-year age categories. YPLL provides an estimate of premature mortality burden by measuring the average time a person would have lived if they had not died prematurely.[2, 5, 6] YPLL due to firearms, age-adjusted YPLL rates due to firearms, and all-cause age-adjusted YPLL rates were exported from WISQARS YPLL Report from 1999 to 2016. YPLL was calculated before the age of 65 because injuries like motor vehicle and firearm injuries are concentrated among younger persons.[6] Stratification variables consisted of sex (male and female), age groups (0–14 years old, 15–44 years old, 45–64 years),[7, 8] race/ethnicity (non-Hispanic white, non-Hispanic black, non-Hispanic American Indian/Alaskan Native, non-Hispanic Asian/Pacific Islander, Hispanic white, Hispanic black, Hispanic American Indian/Alaskan Native, and Hispanic Asian/Pacific Islander), intent (assault/homicide, suicide, unintentional, undetermined, and legal intervention), 50 US states and the District of Columbia. The age categories were selected from previous studies that demonstrated differences in profiles within these age categories.[7, 8] Legal intervention firearm deaths are firearm injuries inflicted by the police or other law-enforcing agents and do not include those deaths caused by firearm injuries by civilians.

### Data analysis

Joinpoint analysis was used to detect changes in temporal patterns of national and state-specific mortality rates, YPLL rates and %YPLL due to firearms as indicated by breakpoints or inflections in the data. Traditionally, assessment of temporal trends is performed using linear regression when a linear trend is observed while piecewise regression may be considered when changes in temporal trends are observed. When analyzing a relationship between response, *y*, and an explanatory variable, *x*, it may be apparent that for different ranges of *x*, different linear relationships occur. In these cases, a single linear model may not provide an adequate description, and a non-linear model may not be appropriate either. Details are presented in S1 Appendix. The analysis was implemented in the Joinpoint Regression Program (V4.1.1, National Cancer Institute).[9, 10] We used age-adjusted mortality rates, and standard errors for the analysis. In the model, the sum of squared errors (SSE) from the model that minimizes SSE, considering *k* joinpoints. For YPLL, the percentage of age-adjusted YPLL rates due to firearms from all-cause age-adjusted YPLL rates was first calculated and then used for analysis. In the Joinpoint Regression Program, the minimum number of observations from a joinpoint to either end of the data was set at two; the minimum number of observations between two joinpoints was set at two; the number of points to place between adjacent observed x values in the grid search was set at zero; and the number of joinpoints was set to range from zero to five based on the number of observations and the pattern visualization conducted prior to specifying the number of joinpoints. The model selection method was set to permutation test with an overall significance level of 0.05 and the number of randomly permuted data sets to be 4499. The auto-correlated errors option was turned off. The confidence intervals of the Annual Percent Change (APC) were set to be parametric. Stratified analyses were also performed based on national, by sex, age group, race/ethnicity, intent, and state. Once we obtained the inflection points, we performed sensitivity analysis using interrupted time series analysis at each inflection point and modeled mortality rates as time series to estimate the effect with pre and post observations.[11]

## Results

### National mortality

**Table 1** details the national- and subgroup-specific trends in age-standardized firearm mortality. Overall, mortality rates were stable between 1999 and 2014, after which there was a significant annual increase of 7.2 percent (95% CI = 3.1, 11.4) through 2016. Firearm mortality rates among men demonstrated a similar pattern, with a significant uptick in 2014 (APC = 6.6, 95% CI = 2.3, 11.0), while female-specific data demonstrated an earlier increase beginning in 2008 (APC = 2.8, 95% CI = 2.0, 3.5). Prior to 2008, there was a significant decrease in mortality among women (APC = -0.8, 95% CI = -1.4, -0.2). The change over the study period for those 15 to 44 (14%) and those 45 to 64 (24%) were different than for those 1 to 14 (0%) and those 65+ (4%) years of age. Adults aged 45 years and older experienced an increase in mortality rate starting in 2006. Younger populations in the 0 to 14 age group did not experience an increase until 2009, at which point the rates increased to 3.8% per year (95% CI = 1.0, 6.8). The largest increase occurred after 2014 in those between 15 and 44 years of age (APC = 10.1, 95% CI = 3.9, 16.8) which followed a slight decline (APC = -0.5, 95% CI -0.7, -0.2) from 1999 to 2014.

**Table 1:** Joinpoint analysis of age-standardized firearm mortality rates in the US between 1999 and 2016.

	Rate per 100,000			Segment 1		Segment 2		Segment 3		Segment 4	
	Total	1999	2016	BP	APC (95% CI)	BP	APC (95% CI)	BP	APC (95% CI)	BP	APC (95% CI)
											
**Overall**	10.5	10.3	11.8	1999–2014	0.03 (-0.2, 0.2)	2014–2016	7.2 (3.1, 11.4)				
											
**By sex**											
Female	2.9	2.9	3.4	1999–2008	-0.8 (-1.4, -0.2)	2008–2016	2.8 (2.0, 3.5)				
Male	18.5	18.4	20.5	1999–2014	-0.1 (-0.3, 0.1)	2014–2016	6.6 (2.3, 11.0)				
											
**By age**											
0–14 years	0.7	0.8	0.8	1999–2009	-1.8 (-3.4, 0.2)	2009–2016	3.8 (1.0, 6.8)				
15–44 years	14.3	14.5	16.5	1999–2014	-0.5 (-0.7, -0.2)	2014–2016	10.1 (3.9, 16.8)				
45–64 years	11.3	10.0	12.4	1999–2002	2.1 (-0.2, 4.6)	2002–2006	-0.1 (-2.3, 2.2)	2006–2010	2.5 (0.3, 4.7)	2010–2016	0.9 (0.2, 1.5)
65+ years	12.3	12.4	12.9	1999–2006	-1.4 (-2.0, -0.8)	2006–2016	1.3 (1.0, 1.7)				
											
**By race/** **ethnicity**											
Non-Hispanic White	9.5	8.9	11.0	1999–2006	-0.4 (-1.0, 0.3)	2006–2016	2.2 (1.8, 2.5)				
Non-Hispanic Black	19.4	19.0	23.0	1999–2006	1.4 (0.3, 2.5)	2006–2009	-4.0 (-11.3, 3.9)	2009–2014	0.0 (-2.7, 2.8)	2014–2016	12.6 (4.8, 21.0)
Non-Hispanic American Indian/ Alaskan Native	11.2	10.7	14.4	1999–2011	2.3 (-1.0, 1.6)	2011–2016	5.9 (1.7, 10.3)				
Non-Hispanic Asian/Pacific Islander	2.7	3.7	2.9	1999–2014	-2.9 (-3.8, -1.9)	2014–2016	14.6 (-4.0, 36.6)				
Hispanic White	7.0	8.5	6.8	1999–2007	-1.1 (-1.7, -0.5)	2007–2010	-6.7 (-10.9, -2.4)	2010–2014	-2.1 (-4.7, 0.6)	2014–2016	8.6 (3.4, 14.1)
Hispanic Black	3.0	3.4	2.9	1999–2016	-0.8 (-1.9, 0.3)						
Hispanic American Indian/ Alaskan Native	1.7	3.1*	2.7*	1999–2016	1.7 (-1.2, 4.7)						
Hispanic Asian/ Pacific Islander	2.5	2.9*	2.9*	1999–2016	-1.1 (-4.2, 2.0)						
											
**By intent**											
Assault/ homicide	4.0	3.8	4.6	1999–2007	1.5 (0.6, 2.4)	2007–2010	-5.4 (-12.7, 2.5)	2010–2014	-0.1 (-4.4, 4.3)	2014–2016	13.2 (5.4, 21.6)
Suicide	6.1	6.0	6.8	1999–2006	-1.1 (-1.6, -0.6)	2006–2016	2.0 (1.6, 2.2)				
Unintentional	0.2	0.3	0.2	1999–2016	-3.3 (-4.6, -1.9)						
Undetermined	0.1	0.1	0.1	1999–2016	-						
Legal intervention	0.1	0.1	0.2	1999–2016	5.4 (3.1, 7.8)						

Rate denotes age-standardized rates per 100,000 persons except for age groups and the non-fatal injuries for race/ethnicity groups. Total denotes cumulative rate. BP denotes year of break point and 2015 represents the end. APC denotes annual percent change, derived from [exp(slope)-1]x100. 95% CI denotes 95% confidence interval.

* denotes rates based on 20 or fewer deaths may be unstable.

Mortality rate increased among non-Hispanic white populations starting in 2006 (APC = 2.2, 95% CI = 1.8, 2.5). Rates among non-Hispanic black populations increased from 1999 to 2006, briefly plateaued, and then increased from 2014 to 2016 at a rate of 12.6 percent (95% CI = 4.8, 21.0). Non-Hispanic American Indian populations experienced an increase in rates after 2011 (APC = 5.9, 95% CI = 1.7, 10.3). Asian/Pacific Islander populations experienced decreasing rates until 2014. The rates increased (APC = 8.6, 95% CI = 3.4, 14.1) from 2014 onwards among Hispanic white populations. The pattern for homicide was similar to that of Non-Hispanic black populations, while suicide rates were comparable to those among non-Hispanic white populations. Unintentional mortality declined while legal intervention rates increased steadily throughout the eighteen years (APC = 5.4, 95% CI = 3.1, 7.8). The largest mortality rate in 2016 was among non-Hispanic black Americans (23.0 per 100,000). **S1, S2, S3, S4 and S5 Figs** presents overall and sub-group specific annual firearm mortality rates.

### National YPLL

**Table 2** details the national- and subgroup-specific trends in age-standardized YPLL. Overall %YPLL rates increased (APC = 0.65, 95% CI = 0.43, 0.87) from 1999 to 2014 and thereafter increased at a faster rate (APC = 5.1, 95% CI = 0.1, 10.4). An increase in %YPLL rates among men occurred between 1999 and 2014 (APC = 0.7, 95% CI = 0.5, 0.9) and between 2004 and 2014 for women (APC = 1.8, 95% CI = 1.1, 2.4), after which the increase plateaued. The decline in %YPLL in non-Hispanic white populations persisted until 2006. After 2006, the data show a breakpoint where the rates among this subgroup began to increase at 3.5 percent per year (95% CI = 2.1, 5.0). A significant increase in %YPLL is shown in Hispanic white populations, with an increase in 2014 of 8.1 percent (95% CI = 2.0, 14.5). This pattern is similar to rates shown among non-Hispanic black populations, which experienced a large increase from 2014 [8.4% (95% CI = 0.7, 16.8)]. A decline was observed among non-Hispanic Asian/Pacific Islander populations during the entire period. %YPLL due to homicide injury, which saw a decline between 2006 and 2014, began to rise annually by 10.2 percent (95% CI = 2.4, 18.8) until 2016. %YPLL due to suicide increased from 2006 onwards at a rate of 4.8% per year (95% CI = 3.8, 5.8), slowing in 2011 to an increase of 1.0 percent (95% CI = 0.4, 1.7). %YPLL related to unintended firearm deaths declined (APC = -3.4, 95% CI = -4.1, -2.7) over the entire period, while legal intervention rates did not change significantly. The largest YPLL rate in 2016 was among non-Hispanic black Americans (860 per 100,000). **S6, S7, S8 and S9 Figs** presents overall and sub-group specific % firearm YPLL.

**Table 2:** Joinpoint analysis of age-standardized rates of years of potential life lost before age 65 related to firearm mortality in the US from 1999 to 2016.

	YPLL Rate per 100,000, (Firearm /ALL YPLL, %)			Segment 1		Segment 2		Segment 3	
	Total	1999	2016	BP	APC (95% CI)	BP	APC (95% CI)	BP	APC (95% CI)
									
**Overall**	299 (6.8)	305 (6.6)	338.2 (7.8)	1999–2014	0.65 (0.43, 0.87)	2014–2016	5.1 (0.1, 10.4)		
											
**By sex**									
Female	82.7 (2.6)	89 (2.6)	88 (2.9)	1999–2004	-2.1 (-3.6, -0.5)	2004–2014	1.8 (1.1, 2.4)	2014–2016	6.2 (-0.6, 13.4)
Male	511 (9.3)	516 (8.9)	572 (10.5)	1999–2014	0.7 (0.5, 0.9)	2014–2016	4.2 (-0.4, 9.1)		
									
**By race/** **ethnicity**									
Non-Hispanic White	231 (5.4)	228 (5.6)	271 (6.3)	1999–2006	-1.5 (-2.0, -0.9)	2006–2011	3.5 (2.1, 5.0)	2011–2016	0.9 (0.0, 1.9)
Non-Hispanic Black	743 (11.0)	730 (8.6)	860 (12.9)	1999–2006	2.7 (1.6, 3.8)	2006–2014	0.9 (-0.2, 1.9)	2014–2016	8.4 (0.7, 16.8)
Non-Hispanic American Indian/ Alaskan Native	382 (5.7)	373 (6.0)	451 (5.8)	1999–2016	-0.1 (-0.9, 0.7)				
Non-Hispanic Asian/Pacific Islander	84 (4.5)	124 (5.4)	88.7 (4.5)	1999–2016	-1.3 (-2.3, -0.3)				
Hispanic White	261 (7.5)	288 (7.2)	231 (7.0)	1999–2006	0.4 (-0.3, 1.1)	2006–2014	-2.5 (-3.2, -1.8)	2014–2016	8.1 (2.0, 14.5)
Hispanic Black	113 (8.2)	133 (8.9)	117 (7.6)	1999–2016	0.8 (-0.1, 1.7)				
Hispanic American Indian/ Alaskan Native	58 (8.2)	116 (11.6)*	85 (10.8)*	1999–2016	-0.6 (03.1, 1.9)				
Hispanic Asian/ Pacific Islander	76 (5.0)	36 (2.8)*	86 (5.4)*	1999–2016	1.7 (-0.5, 4.0)				
									
**By intent**									
Assault/ homicide	153 (3.5)	150 (3.3)	174 (4.0)	1999–2006	2.0 (1.0, 3.1)	2006–2014	-1.2 (-2.3, -0.2)	2014–2016	10.2 (2.4, 18.8)
Suicide	131 (3.0)	136 (3.0)	150 (3.5)	1999–2006	-1.7 (-2.1, -1.3)	2006–2011	4.8 (3.8, 5.8)	2011–2016	1.0 (0.4, 1.7)
Unintentional	7 (0.2)	11 (0.2)	6 (0.1)	1999–2016	-3.4 (-4.1, -2.7)				
Undetermined	3 (0.1)	4 (0.0)	3 (0.0)	1999–2001	-19.5 (-35.1, -0.1)	2001–2016	1.1 (0.1, 2.2)		
Legal intervention	4 (0.1)	4 (8.2)	5 (0.1)	1999–2009	0.8 (-0.6, 2.3)	2009–2012	12.7 (-5.9, 34.9)	2012–2016	-1.4 (-6.3, 3.8)

Rate denotes age-standardized rates per 100,000 persons except for age groups and the non-fatal injuries for race/ethnicity groups. Total denotes cumulative rate. BP denotes year of break point and 2015 represents the end. APC denotes annual percent change, derived from [exp(slope)-1]x100. BP denotes year of break point and 2015 represents the end. APC denotes annual percent change, derived from [exp(slope)-1]x100. YPLL denotes Years of Potential Life Lost at 65 years. YPLL Rate denotes age-adjusted YPLL rates per 100,000 persons. % is 100x(YPLL due to firearm/ YPLL due to all causes). 95% CI denotes 95% confidence interval.

* denotes rates based on 20 or fewer deaths may be unstable.

### State mortality

**Fig 1:**
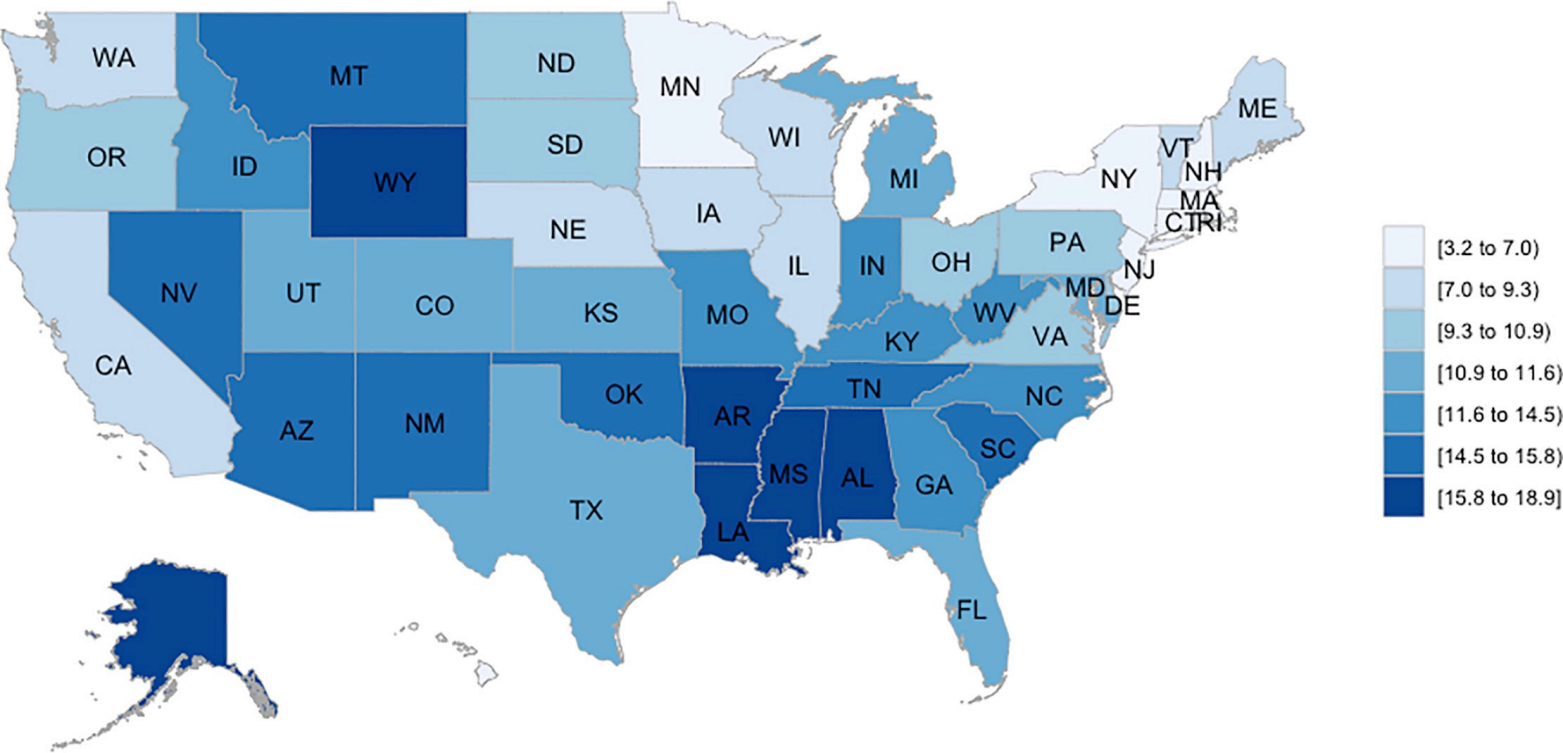
Cumulative firearm age-adjusted mortality rates per 100,000 from 1999 to 2016.

The spatial differences in cumulative age-adjusted mortality rates from 1999 to 2016 are presented in **Fig 1**. During the entire period, Hawaii had the lowest cumulative age-adjusted rates (3.2 per 100,000) while Louisiana had the highest (18.9 per 100,000). The lowest annual mortality rate during the study period was in Hawaii in 2005 (2.1 per 100,000), and the highest was in Alaska in 2016 (23.3 per 100,000). **Table 3** details the national- and subgroup-specific trends in age-standardized firearm mortality by state. Mortality rates declined steadily from 1999 to 2016 in New York and Arizona. Conversely, twenty-two states saw a steady increase in mortality throughout this time: New Hampshire, Vermont, New Jersey, Pennsylvania, Michigan, Ohio, Iowa, Kansas, Minnesota, North Dakota, South Dakota, Delaware, Florida, West Virginia, Mississippi, Arkansas, Louisiana, Idaho, Montana, Utah, Wyoming, and Alaska. Thirteen states (Maine, Illinois, Indiana, Wisconsin, Missouri, Nebraska, Georgia, Maryland, South Carolina, Oklahoma, Nevada, New Mexico, and Oregon), which saw either declining or steady rates starting in 1999, began to experience an increase in rate at some point within the eighteen-year period, although there was no distinct pattern to this change when compared between states. No significant change was observed between 1999 and 2016 in eight states (Connecticut, Massachusetts, Rhode Island, Alabama, Kentucky, Tennessee, Texas, and Washington). Rates in North Carolina, Virginia, California, Hawaii, and the District of Columbia remained steady throughout the eighteen years; however, each experienced a decline in firearm-related mortality at one point before returning to a steady rate. Two states, Illinois (APC = 14.0, 95% CI = 5.2, 25.3) and Missouri (APC = 12.6, 95% CI = 2.6, 23.6), experienced annual increases >10% in their firearm-related mortality rates from 2014 to 2016. **S10, S11, S12 and S13 Figs** presents state-specific firearm mortality rates across time.

**Table 3:** Joinpoint analysis of age-standardized firearm mortality rates in the US states from 1999 to 2016.

	Rate per 100,000			Segment1		Segment 2		Segment 3		Segment 4	
	Total	1999	2016	BP	APC (95% CI)	BP	APC (95% CI)	BP	APC (95% CI)	BP	APC (95% CI)
**R1: North-east**											
**D1: New England**											
Connecticut	5.1	5.9	4.6	1999–2016	0.04 (-1.2, 1.3)						
Maine	9.1	8.7	8.3	1999–2002	-8.3 (-20.7, 6.0)	2002–2016	2.9 (1.4, 4.3)				
Massachusetts	3.3	3.0	3.4	1999–2016	0.8 (-0.2, 1.8)						
New Hampshire	7.1	5.8	9.3	1999–2016	2.3 (1.1, 3.6)						
Rhode Island	4.2	5.3	4.1	1999–2016	-0.7 (-2.5, 1.1)						
Vermont	9.3	9.3	11.1	1999–2016	1.2 (0.1, 2.4)						
											
**D2: Middle Atlantic**											
New Jersey	5.1	4.4	5.5	1999–2016	1.1 (0.4, 1.8)						
New York	4.8	5.3	4.4	1999–2016	-1.8 (-2.2, -1.3)						
Pennsylvania	10.6	9.7	12.0	1999–2016	1.0 (0.6, 1.3)						
											
**R2: Midwest**											
**D3: East North Central**											
Illinois	9.1	9.7	11.7	1999–2001	4.3 (-4.6, 14.0)	2001–2004	-7.9 (-14.8, 0.7)	2004–2014	1.0 (0.1, 2.0)	2014–2016	14.0 (5.2, 23.5)
Indiana	11.8	11.2	15.0	1999–2011	-0.4 (-1.3, 0.5)	2011–2016	5.7 (2.4, 9.1)				
Michigan	11.2	11.0	12.3	1999–2016	0.6 (0.3, 1.0)						
Ohio	9.9	8.2	12.9	1999–2016	2.3 (1.7, 2.9)						
Wisconsin	8.6	8.3	11.4	1999–2011	-0.4 (-1.6, 0.9)	2011–2016	6.6 (2.0, 11.4)				
											
**D4: West North Central**											
Iowa	7.0	6.8	9.2	1999–2016	1.5 (0.5, 2.4)						
Kansas	11.1	10.0	13.4	1999–2016	1.1 (0.4, 1.8)						
Minnesota	6.9	6.1	7.6	1999–2016	0.9 (0.4, 1.5)						
Missouri	14.3	12.7	19.0	1999–2003	-2.8 (-6.3, 0.8)	2003–2014	2.4 (1.6, 3.2)	2014–2016	12.6 (2.6, 23.6)		
Nebraska	8.3	8.6	9.1	1999–2004	-4.7 (-8.5, -0.7)	2004–2016	2.3 (1.2, 3.3)				
North Dakota	9.5	7.5	11.9	1999–2016	3.4 (2.3, 4.4)						
South Dakota	9.6	9.7	13.4	1999–2016	1.8 (0.4, 3.2)						
											
**R3: South**											
**D5: South Atlantic**											
Delaware	9.6	8.7	11.0	1999–2016	2.0 (1.1, 3.0)						
District of Columbia	18.0	25.5	13.8	1999–2002	5.0 (-8.2, 20.1)	2002–2008	-5.6 (-11.4, 0.5)	2008–2012	-16.9 (-30.7, -0.3)	2012–2016	12.6 (-0.3, 27.2)
Florida	11.5	10.7	12.6	1999–2016	1.0 (0.5, 1.4)						
Georgia	13.2	13.7	15.0	1999–2013	-0.5 (-1.0, 0.0)	2013–2016	6.3 (1.0, 11.8)				
Maryland	11.1	12.7	11.9	1999–2007	-0.0 (-2.1, 2.0)	2007–2011	-7.0 (-15.8, 2.7)	2011–2016	5.4 (1.0, 10.0)		
North Carolina	12.5	13.1	13.7	1999–2014	-1.0 (-1.3, -0.6)	2014–2016	8.8 (-0.2, 18.6)				
South Carolina	14.6	13.8	17.7	1999–2009	-0.1 (-1.0, 0.7)	2009–2016	3.8 (2.4, 5.2)				
Virginia	10.8	11.7	12.1	1999–2014	-0.7 (-1.1, -0.3)	2014–2016	9.0 (-0.4, 19.4)				
West Virginia	14.3	13.3	17.5	1999–2016	0.9 (0.2, 1.5)						
											
**D6: East South Central**											
Alabama	17.4	17.7	21.5	1999–2014	0.1 (-0.5, 0.7)	2014–2016	13.4 (-0.1, 28.8)				
Kentucky	13.8	12.9	17.5	1999–2014	0.5 (-0.2, 1.1)	2014–2016	12.6 (-0.6, 27.5)				
Mississippi	17.8	18.2	19.9	1999–2016	0.7 (0.1, 1.2)						
Tennessee	15.4	14.8	17.1	1999–2014	-0.03 (-0.5, 0.5)	2014–2016	6.7 (-3.8, 18.4)				
											
**D7: West South Central**											
Arkansas	16.0	14.6	17.8	1999–2016	0.8 (0.4, 1.2)						
Louisiana	19.0	17.5	21.3	1999–2016	0.6 (0.3, 1.0)						
Oklahoma	14.9	14.8	19.6	1999–2005	-1.9 (-4.5, 0.8)	2005–2016	3.6 (2.6, 4.5)				
Texas	11.0	10.5	12.1	1999–2014	-0.1 (-0.4, 0.2)	2014–2016	6.7 (-0.6, 14.5)				
											
**R4: West**											
**D8: Mountain**											
Arizona	15.1	16.3	15.2	1999–2016	-1.1 (-1.6, -0.5)						
Colorado	11.6	10.8	14.3	1999–2011	-0.1 (-1.2, 1.1)	2011–2016	4.5 (0.8, 8.3)				
Idaho	13.2	12.2	14.6	1999–2016	0.9 (0.2, 1.6)						
Montana	16.1	13.4	18.9	1999–2016	1.3 (0.4, 2.2)						
Nevada	15.7	20.0	16.8	1999–2001	-7.2 (-14.7, 0.9)	2001–2007	-0.8 (-2.5, 1.0)	2007–2012	-4.2 (-6.6, -1.7)	2012–2016	5.9 (3.4, 8.5)
New Mexico	15.9	16.3	18.1	1999–2011	-1.1 (-2.0, -0.1)	2011–2016	5.1 (1.6, 8.8)				
Utah	11.3	9.4	12.9	1999–2016	1.8 (1.2, 2.4)						
Wyoming	15.2	15.2	17.4	1999–2016	1.5 (0.2, 2.9)						
											
**D9: Pacific**											
Alaska	19.0	15.8	23.3	1999–2016	1.5 (0.4, 2.5)						
California	8.5	9.3	7.9	1999–2005	0.5 (-1.3, 2.2)	2005–2010	-4.2 (-6.6, -1.7)	2010–2016	-0.1 (-1.2, 0.9)		
Hawaii	3.1	3.4	4.5	1999–2006	-6.8 (-12.4, -1.0)	2006–2011	8.7 (-5.6, 25.3)	2011–2014	-11.2 (-42.0, 36.1)	2014–2016	34.3 (-8.7, 97.5)
Oregon	10.8	11.3	11.9	1999–2007	-1.1 (-2.3, 0.2)	2007–2016	1.8 (0.9, 2.8)				
Washington	9.2	10.1	9.0	1999–2016	0.04 (-0.5, 0.6)						

Rate denotes age-standardized rates per 100,000 persons except for age groups and the non-fatal injuries for race/ethnicity groups. Total denotes cumulative rate. BP denotes year of break point and 2015 represents the end. BP denotes year of break point and 2015 represents the end. APC denotes annual percent change, derived from [exp(slope)-1]x100. 95% CI denotes 95% confidence interval.

### State YPLL

**Fig 2:**
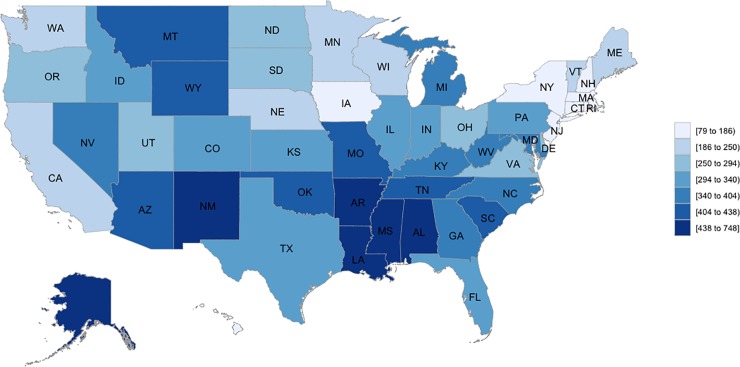
Cumulative firearm age-adjusted years of potential life lost rates per 100,000 from 1999 to 2016.

The spatial differences in cumulative age-adjusted YPLL rates from 1999 to 2016 are presented in **Fig 2**. During the entire period, Hawaii had the lowest cumulative age-adjusted YPLL rates (79.0 per 100,000) while District of Columbia had the highest (750.4 per 100,000). The %YPLL due to firearm deaths was also lowest in Hawaii (2.1%) and highest in the District of Columbia (13.2%). The lowest age-adjusted YPLL rate was in Hawaii in 2014 (51.4 per 100,000) Tand the highest was in District of Columbia in 2002 (1272.7 per 100,000). **Table 4** details the age-standardized YPLL in 1996 and 2016 and proportion of YPLL due to firearms from overall deaths by state. Between 1999 and 2016, %YPLL rates declined steadily in Rhode Island, the District of Columbia, and Arizona. A steady increase in %YPLL across the entire period was observed in Pennsylvania, Michigan, Ohio, Iowa, Kansas, Minnesota, North Dakota, South Dakota, Delaware, Louisiana, Texas, Idaho, Utah, Wyoming, and Alaska. Eight states exhibited no significant change (New Hampshire, Vermont, Maryland, West Virginia, Alabama, Kentucky, Arkansas, and Montana). Several states (Illinois, Indiana, Wisconsin, Missouri, Nebraska, Georgia, North Carolina, South Carolina, Mississippi, Tennessee, Oklahoma, Colorado, Nevada, Oregon, and Washington) which began with either a declining or steady %YPLL in 1999, experienced an increase in rate after the initial decline. **S14, S15, S16 and S17 Figs** presents state-specific firearm % YPLL across time. Sensitivity analysis using interrupted time series analysis confirmed the inflection points (**S18 Fig**).

**Table 4:** Joinpoint analysis age-standardized rates of years of potential life lost before age 65 related to firearm mortality in the US states from 1999 to 2016.

	Firearm/ALL YPLL (%)			Segment1		Segment 2		Segment 3		Segment 4	
	Total	1999	2016	BP	APC (95% CI)	BP	APC (95% CI)	BP	APC (95% CI)	BP	APC (95% CI)
**R1: North-east**											
**D1: New England**											
Connecticut	147 (4.2)	182 (4.9)	123.2 (3.6)	1999–2012	2.7 (0.5, 5.0)	2012–2016	-9.9 (-22.3, 4.4)				
Maine	202 (4.9)	226.6 (6.2)	214.2 (4.8)	1999–2002	-14.7 (-27.2, 0.1)	2002–2013	3.9 (1.3, 6.6)	2013–2016	-7.4 (-20.1, 7.4)		
Massachusetts	101 (3.0)	90.4 (2.6)	99.4 (2.8)	1999–2010	4.0 (2.6, 5.5)	2010–2016	-6.4 (-9.5, -3.2)				
New Hampshire	172 (4.9)	161.4 (4.8)	235.0 (5.4)	1999–2016	1.6 (-0.1, 3.3)						
Rhode Island	126 (3.5)	171.1 (4.9)	94.7 (2.5)	1999–2016	-2.0 (-3.7, -0.3)						
Vermont	216 (6.0)	237.1 (6.5)	257.9 (6.7)	1999–2016	1.4 (-0.1, 2.9)						
											
**D2: Middle Atlantic**											
New Jersey	168 (4.3)	133.8 (3.2)	181.3 (5.1)	1999–2005	8.5 (4.8, 12.4)	2005–2016	0.6 (-0.6, 1.9)				
New York	153 (4.3)	178.7 (4.3)	127.4 (3.8)	1999–2011	0.3 (-0.6, 1.2)	2011–2016	-4.3 (-8.0, -0.6)				
Pennsylvania	320 (7.0)	309.8 (6.8)	354.1 (7.4)	1999–2016	0.7 (0.3, 1.1)						
											
**R2: Midwest**											
**D3: East North Central**											
Illinois	305 (7.2)	336.9 (7.0)	402.2 (9.7)	1999–2001	4.0 (-4.0, 12.6)	2001–2004	-6.3 (-13.5, 1.6)	2004–2014	2.5 (1.7, 3.4)	2014–2016	10.6 (2.6, 19.3)
Indiana	343 (7.1)	347.2 (7.3)	435.0 (8.4)	1999–2011	-0.9 (-1.7, -0.1)	2011–2016	5.5 (2.6, 8.5)				
Michigan	339 (7.2)	350.8 (7.3)	362.6 (7.6)	1999–2016	0.6 (0.2, 1.0)						
Ohio	287 (5.9)	225.5 (4.9)	377.6 (7.0)	1999–2016	2.0 (1.5, 2.6)						
Wisconsin	249 (6.4)	261.6 (6.7)	342.0 (8.5)	1999–2011	-0.6 (-1.9, 0.8)	2011–2016	6.7 (1.9, 11.7)				
											
**D4: West North Central**											
Iowa	187 (5.1)	176.5 (4.7)	259.8 (6.6)	1999–2016	1.5 (0.4, 2.7)						
Kansas	312 (7.2)	297.1 (6.7)	384.5 (9.2)	1999–2016	1.3 (0.3, 2.2)						
Minnesota	188 (5.7)	178.6 (5.1)	208.8 (6.5)	1999–2016	1.1 (0.4, 1.9)						
Missouri	418 (8.3)	382.3 (7.6)	578.5 (11.2)	1999–2003	-5.1 (-11.1, 1.2)	2003–2016	3.7 (2.7, 4.7)				
Nebraska	235 (6.2)	260.6 (6.4)	272.1 (7.5)	1999–2002	-8.7 (-19.4, 3.4)	2002–2016	2.6 (1.4, 3.8)				
North Dakota	268 (6.9)	232.6 (5.9)	318.3 (7.5)	1999–2016	2.0 (0.8, 3.1)						
South Dakota	271 (5.9)	300.1 (6.2)	392.5 (8.5)	1999–2016	1.7 (0.2, 3.3)						
											
**R3: South**											
**D5: South Atlantic**											
Delaware	289 (5.9)	267.8 (5.8)	313.0 (6.5)	1999–2016	2.7 (1.2, 4.2)						
District of Columbia	736 (13.1)	1133.1 (12.5)	515.4 (10.4)	1999–2016	-2.0 (-3.3, -0.7)						
Florida	314 (6.5)	297.8 (5.9)	356.6 (7.6)	1999–2005	0.2 (-2.2, 2.5)	2005–2008	8.3 (-4.9, 23.3)	2008–2016	1.0 (-0.5, 2.4)		
Georgia	368 (7.5)	388.5 (7.2)	447.8 (9.2)	1999–2005	-1.8 (-3.5, -0.1)	2005–2016	3.1 (2.4, 3.7)				
Maryland	371 (7.9)	458.5 (9.0)	394.2 (8.6)	1999–2016	-0.3 (-1.1, 0.4)						
North Carolina	350 (7.2)	386.0 (7.3)	391.9 (8.2)	1999–2009	-0.6 (-1.3, 0.1)	2009–2016	1.9 (0.6, 3.3)				
South Carolina	418 (7.4)	405.56 (6.7)	509.9 (9.4)	1999–2007	1.0 (-0.3, 2.3)	2007–2016	5.6 (2.5, 4.7)				
Virginia	295 (7.2)	321.9 (7.5)	333.4 (8.5)	1999–2013	-0.0 (-0.7, 0.6)	2013–2016	5.8 (-1.2, 13.2)				
West Virginia	366 (5.9)	344.3 (6.7)	465.4 (6.9)	1999–2016	-0.2 (-1.0, 0.5)						
											
**D6: East South Central**											
Alabama	498 (7.9)	506.5 (8.0)	632.6 (9.8)	1999–2014	0.3 (-0.5, 1.0)	2014–2016	12.4 (-3.3, 30.6)				
Kentucky	361 (6.4)	344.8 (6.9)	483.8 (7.9)	1999–2014	-0.3 (-1.1, 0.6)	2014–2016	11.2 (-6.3, 32.1)				
Mississippi	520 (7.7)	528.6 (7.7)	617.6 (9.5)	1999–2005	-2.0 (-5.2, 1.2)	2005–2016	2.5 (1.2, 3.8)				
Tennessee	426 (7.4)	437.8 (7.8)	486.0 (8.6)	1999–2003	-3.4 (-6.7, 0.0)	2003–2016	1.3 (0.7, 1.9)				
											
**D7: West South Central**											
Arkansas	444 (7.4)	420.3 (7.4)	478.2 (8.1)	1999–2016	0.7 (-0.0, 1.3)						
Louisiana	605 (9.7)	538.4 (8.5)	668.78 (11.0)	1999–2016	1.2 (0.8, 1.5)						
Oklahoma	410 (7.1)	441.7 (7.9)	539.28 (9.3)	1999–2004	-5.0 (-8.8, -1.0)	2004–2016	3.4 (2.4, 4.5)				
Texas	298 (6.9)	288.9 (6.3)	339.6 8.4	1999–2014	0.6 (0.3, 0.9)	2014–2016	9.8 (2.3, 17.8)				
											
**R4: West**											
**D8: Mountain**											
Arizona	412 (9.0)	463.6 (9.5)	386.3 (8.7)	1999–2016	-0.8 (-1.4, -0.2)						
Colorado	298 (7.8)	297.0 (7.3)	380.9 (10.0)	1999–2008	-0.3 (-2.0, 1.5)	2008–2016	4.0 (2.0, 5.9)				
Idaho	323 (8.0)	306.2 (7.3)	358.6 (9.0)	1999–2016	1.6 (0.7, 2.5)						
Montana	422 (8.7)	349.5 (7.8)	523.0 (10.7)	1999–2016	1.0 (-0.3, 2.3)						
Nevada	394 (8.3)	487.7 (9.7)	429.08 (9.5)	1999–2013	-1.4 (-1.9, -1.0)	2013–2016	7.7 (2.6, 13.1)				
New Mexico	444 (8.2)	500.8 (9.4)	502.7 (8.5)	1999–2006	-3.6 (-6.2, -1.0)	2006–2016	1.4 (-0.2, 3.0)				
Utah	279 (7.4)	245.5 (6.3)	325.5 (8.3)	1999–2016	2.0 (1.2, 2.8)						
Wyoming	407 (8.3)	379.0 (7.9)	385.3 (8.4)	1999–2016	2.1 (0.4, 3.6)						
											
**D9: Pacific**											
Alaska	582 (11.5)	469.2 (9.9)	796.5 (14.6)	1999–2016	1.6 (0.4, 2.8)						
California	246 (7.2)	267.4 (6.9)	222.4 (6.9)	1999–2005	1.7 (0.4, 3.1)	2005–2014	-2.0 (-3.0, -1.1)	2014–2016	4.8 (-4.2, 14.5)		
Hawaii	78 (2.1)	91.7 (2.4)	134.7 (3.7)	1999–2006	-8.8 (-15.2, -1.9)	2006–2011	11.3 (-7.4, 33.8)	2011–2014	-16.5 (-53.7, 50.6)	2014–2016	57.6 (-1.8, 152.8)
Oregon	252 (6.5)	275.5 (6.9)	275.6 (7.6)	1999–2003	-4.8 (-9.0, -0.4)	2003–2016	2.3 (1.6, 3.1)				
Washington	230 (6.5)	263.1 (7.1)	231.6 (7.0)	1999–2001	-10.0 (-22.7, 4.8)	2001–2016	1.8 (1.1, 2.4)				

Rate denotes age-standardized rates per 100,000 persons except for age groups and the non-fatal injuries for race/ethnicity groups.

BP denotes year of break point and 2015 represents the end. Total denotes cumulative rate. BP denotes year of break point and 2015 represents the end. APC denotes annual percent change, derived from [exp(slope)-1]x100. YPLL denotes Years of Potential Life Lost at 65 years. YPLL Rate denotes age-adjusted YPLL rates per 100,000 persons. % is 100x(YPLL due to firearm/ YPLL due to all causes). 95% CI denotes 95% confidence interval. Rates based on 20 or fewer deaths may be unstable.

## Discussion

National firearm mortality rates in the United States remained stable between 1999 and 2014, followed by an increase from 2014 to 2016. In contrast, the national %YPLL demonstrated a small annual increase until 2014, followed by an increase, 5-times that before 2014. This early increase in %YPLL which began in 1999, with a significant upward inflection in 2014 was also observed in population subgroups and individual states, suggesting a non-linear pattern that does not traditionally appear in the assessment of temporal trends of firearm-related mortality burden. A constant mortality rate with increasing %YPLL indicates that although the magnitude of firearm deaths remained the same, the deaths were increasingly premature or among younger people across time. There are four additional observations. *First*, the rise in mortality rate after 2014 observed in the overall national rates was driven principally by increases among men, non-Hispanic black populations, Hispanic white populations, and due to homicide. *Second*, mortality rates increased in twenty-one states throughout the time: thirteen states with increases toward the latter part of the eighteen years. Only two states demonstrated a decline. *Third*, an increasing %YPLL in states with increasing mortality rates indicates that there may be a shift in mortality burden driven by the deaths of young Americans. This shift was observed in fourteen states. *Fourth*, stable firearm mortality rates were observed in the company of increasing %YPLL rates in Tennessee, Texas, and Washington, also indicating a shift in burden towards younger people.

To the best of our knowledge, the co-occurrence of an increase in mortality rates beginning in 2014 coupled with a more substantial increase in YPLL from 2014 onwards after a moderate increase from 1999 onwards has never been reported. We used YPLL before 65 years of age as a public health measure used by the Centers for Disease Control and Prevention with the conventional cut-off year at 65 years to quantify the impact of deaths from injuries.[12] This pattern of firearm mortality burden before 2014 is in line with previous studies that have assessed temporal trends in firearm mortality.[1, 3, 13] Recent 2017 firearm death rates (12 per 100,000) indicate that the upward inflection from 2014 has continued beyond 2016.[2] The pre-2014 plateau in mortality among men and homicide deaths was also observed in earlier studies using national data,[3, 14] while state-specific assessment has demonstrated increasing firearm homicide rates and a connected difference in these rates by race.[15, 16] The plateau among non-Hispanic black populations and Hispanic white populations for all has not been previously reported, primarily due to lack of reliable estimates and information on populations of Hispanic ethnicity in other datasets. The documented upward firearm death rate trends that began in 2014 within these subgroups are novel observations and indicate that these four subgroups carry the burden of firearm deaths disproportionately. We also found that the increasing trends in YPLL began as early as 2006, indicating that the burden of firearm mortality shifted to younger Americans from 2006. Future studies should study the impact of laws in separate periods and the inflection point in firearm mortality that may be associated with the passage of firearm-related laws. The corresponding increase in suicides starting in 2006 in both mortality and %YPLL is noteworthy and signifies a shift in the burden of suicide firearm mortality to younger persons, particularly within non-Hispanic white populations.

Twenty-one states experienced an increase in rates of mortality due to firearm injury between 1999 and 2016. Several states also saw inflections followed by an increase in mortality rates later in these eighteen years. Another study analyzed trends from 2000 to 2010 and found spatial heterogeneity in the data.[3] This study reported that only Massachusetts and Florida showed an increasing trend.[3] Our study adds 34 states to this list. This change is accounted for by the addition of six years of mortality data. Additionally, we report a declining trend in New York and Arizona. An earlier study reported seven states, of which New York is one, and the District of Columbia on a declining trend. Together with our results, this confirms a sustained decline in New York. The decline in both mortality and YPLL rates in Arizona is a novel finding and may be accounted for by a reduction in firearm violence and the addition of 44 trauma centers since 2015 for treatment of firearm injury in that state.[17]

A mortality shift towards young persons was observed in fourteen states, where these states had stable mortality rates but increasing YPLL rates, which may be due to deaths among younger people that are either firearm homicide-related or due to suicide. This change in the burden of firearm mortality is of significance to public health and indicates that findings regarding younger individuals need to be considered in any policy solutions considered. The recent rising national trend in firearm deaths is of public health concern. Although state-specific heterogeneity in firearm mortality has been shown before, the presence of periods where changes occurred that may be due to changes in the implementation of new and existing state laws or violence prevention programs remains undocumented.

In recent years, the main approach to reducing firearm violence has been the implementation of state laws to restrict gun ownership. Our results suggest increasing firearm deaths despite the implementation of these new gun laws, suggesting that approaches that take into account the context of firearm homicides more fully may better lead to a mitigation of the consequences of guns. Studies to assess comprehensive solutions firearm related violence are also needed.

Our study has several limitations. First, firearm deaths are coded as a secondary diagnosis that may lead to potential under-reporting of such events. The death records can be coded erroneously,[18], and the intentionality behind firearm deaths can be misclassified.[19] We obtained aggregate data which does not hold information to assess this inconsistency. Second, misclassification of firearm deaths may also be due to racial differences resulting in a more significant number of events among minority population groups and may present as misclassification bias which may likely be more uniform due to use of standardized measures used for collecting death information. However, we cannot mitigate this bias in our study. We used aggregate data from WISQARS to obtain estimates, and we are unable to use enough variables for performing multiple imputation in case of missing data on race. Third, although homicide and suicide are distinct categories of firearm deaths within states, annual counts in a few states were below 10 which would prevent analysis and reporting. However, our results point to trend patterns which will inform future studies. Fourth, there are wide variations in firearm death rates within each state, which may be due to varying county-level estimates. We do not consider county-level data since firearm laws are implemented at the state level with some changes at the county level. However, the social- and community-level characteristics which may drive such changes are not addressed here.

## Conclusion

Between 1999 and 2016, the national rates of firearm mortality and YPLL indicating a shift in the burden of mortality towards younger individuals. While an increase in mortality was noted starting in 2014, the change towards an increase was set much earlier in different subgroups and states. Additional studies to examine county-specific patterns and the factors that explain the differences in trajectories is needed. Future interventions, programs, and policies should be created to address this shifting burden locally and should bear in mind the populations that are being most affected by shifts in firearm death.

## Supporting information

S1 Checklist STROBE Statement—Checklist of items that should be included in reports of cross-sectional studies. (DOCX)

S1 Fig National Firearm mortality rates across time, 1999–2016. (PDF)

S2 Fig National Firearm mortality rates by sex across time, 1999–2016. (PDF)

S3 Fig National Firearm mortality rates by age groups across time, 1999–2016. (PDF)

S4 Fig National Firearm mortality rates by race/ethnicity across time, 1999–2016. (PDF)

S5 Fig National Firearm mortality rates by intent across time, 1999–2016. (PDF)

S6 Fig National all-cause and Firearm YPLL across time, 1999–2016. (PDF)

S7 Fig National Firearm %YPLL by sex across time, 1999–2016. (PDF)

S8 Fig National Firearm %YPLL by race/ethnicity across time, 1999–2016. (PDF)

S9 Fig National Firearm %YPLL by intent across time, 1999–2016. (PDF)

S10 Fig State-specific Firearm mortality rates in North-east region across time, 1999–2016. (PDF)

S11 Fig State-specific Firearm mortality rates in Midwest region across time, 1999–2016. (PDF)

S12 Fig State-specific Firearm mortality rates in South region across time, 1999–2016. (PDF)

S13 Fig State-specific Firearm mortality rates in West region across time, 1999–2016. (PDF)

S14 Fig State-specific Firearm %YPLL in North-east region across time, 1999–2016. (PDF)

S15 Fig State-specific Firearm %YPLL in Midwest region across time, 1999–2016. (PDF)

S16 Fig State-specific Firearm %YPLL in South region across time, 1999–2016. (PDF)

S17 Fig State-specific Firearm %YPLL in west region across time, 1999–2016. (PDF)

S18 Fig Sensitivity analysis using interrupted time series analysis. (PDF)

S1 Appendix Statistical appendix. (DOCX)
