# Supplementary figures and images for: Changes in patterns of mortality rates and years of life lost due to firearms in the United States, 1999 to 2016: A joinpoint analysis
Source: PLoS One. 2019 Nov 22;14(11):e0225223. doi: 10.1371/journal.pone.0225223 (PMC6874349; doi:10.1371/journal.pone.0225223)

## Supplementary Appendix

**S1 Fig: National Firearm mortality rates across time, 1999-2016**

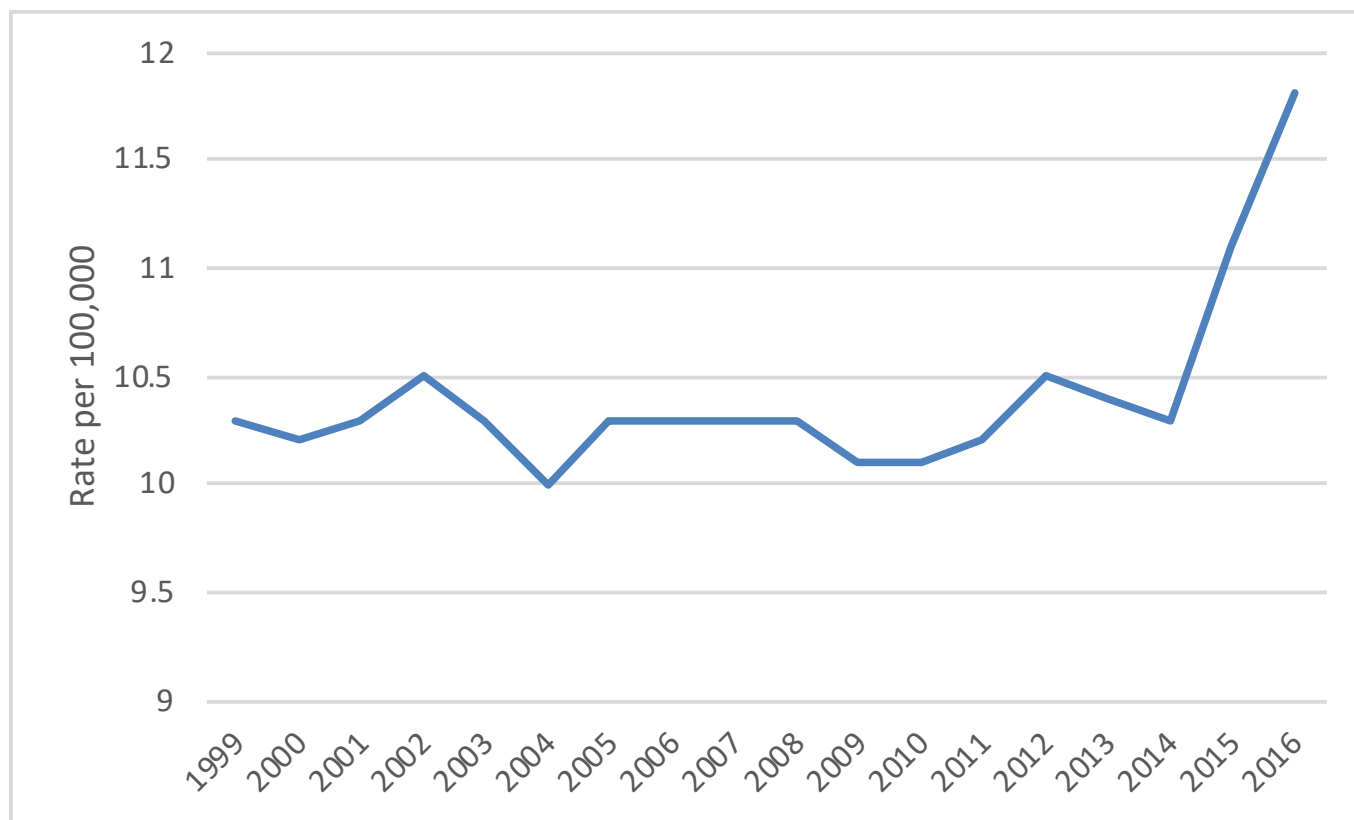

Supplement: S1 Fig — (PDF) [file pone.0225223.s002.pdf]

## Supplementary Appendix

**S2 Fig: National Firearm mortality rates by sex across time, 1999-2016**

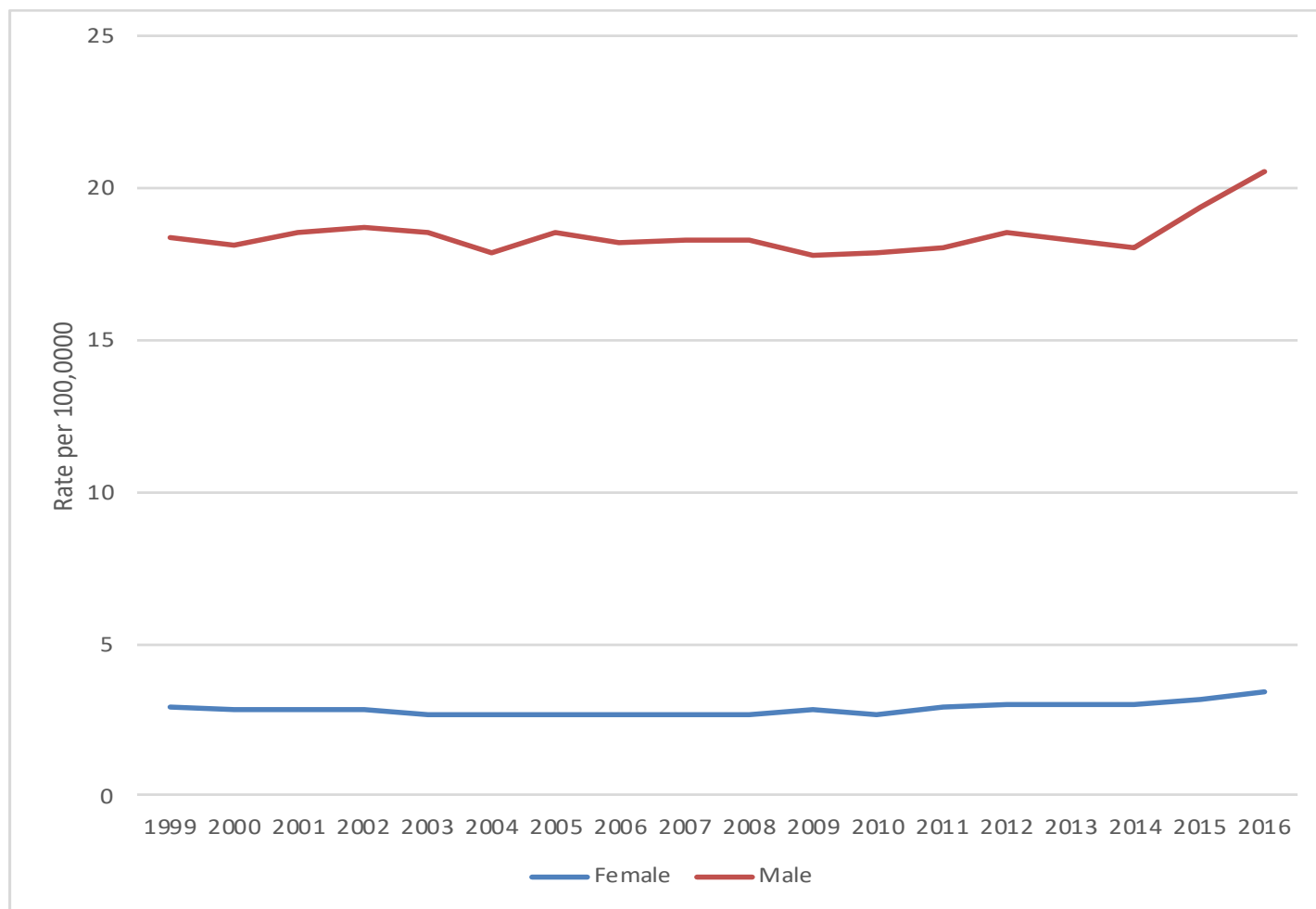

Supplement: S2 Fig — (PDF) [file pone.0225223.s003.pdf]

## Supplementary Appendix

**S3 Fig: National Firearm mortality rates by age groups across time, 1999-2016**

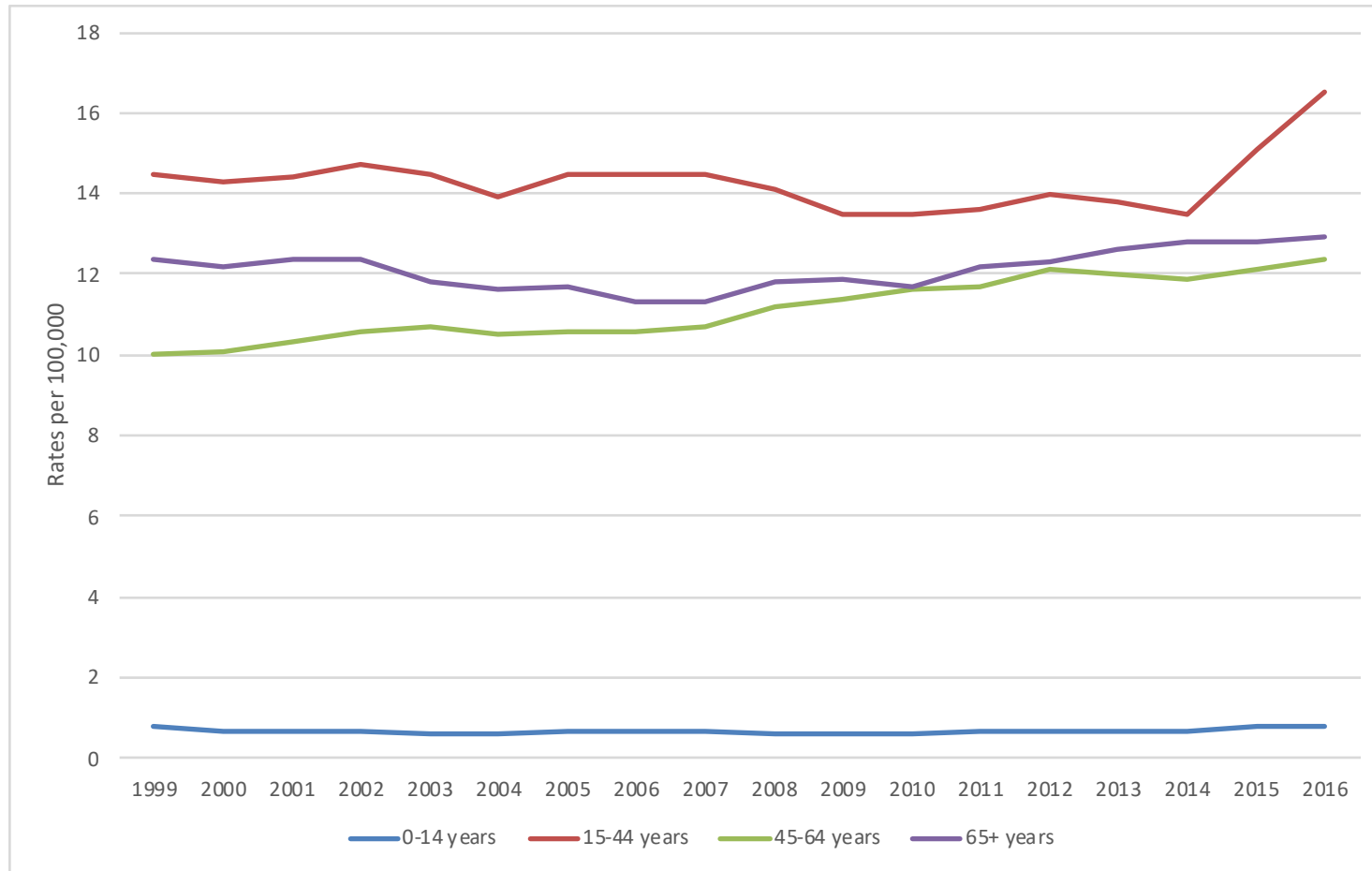

Supplement: S3 Fig — (PDF) [file pone.0225223.s004.pdf]

## Supplementary Appendix

**S4 Fig: National Firearm mortality rates by race/ethnicity across time, 1999-2016**

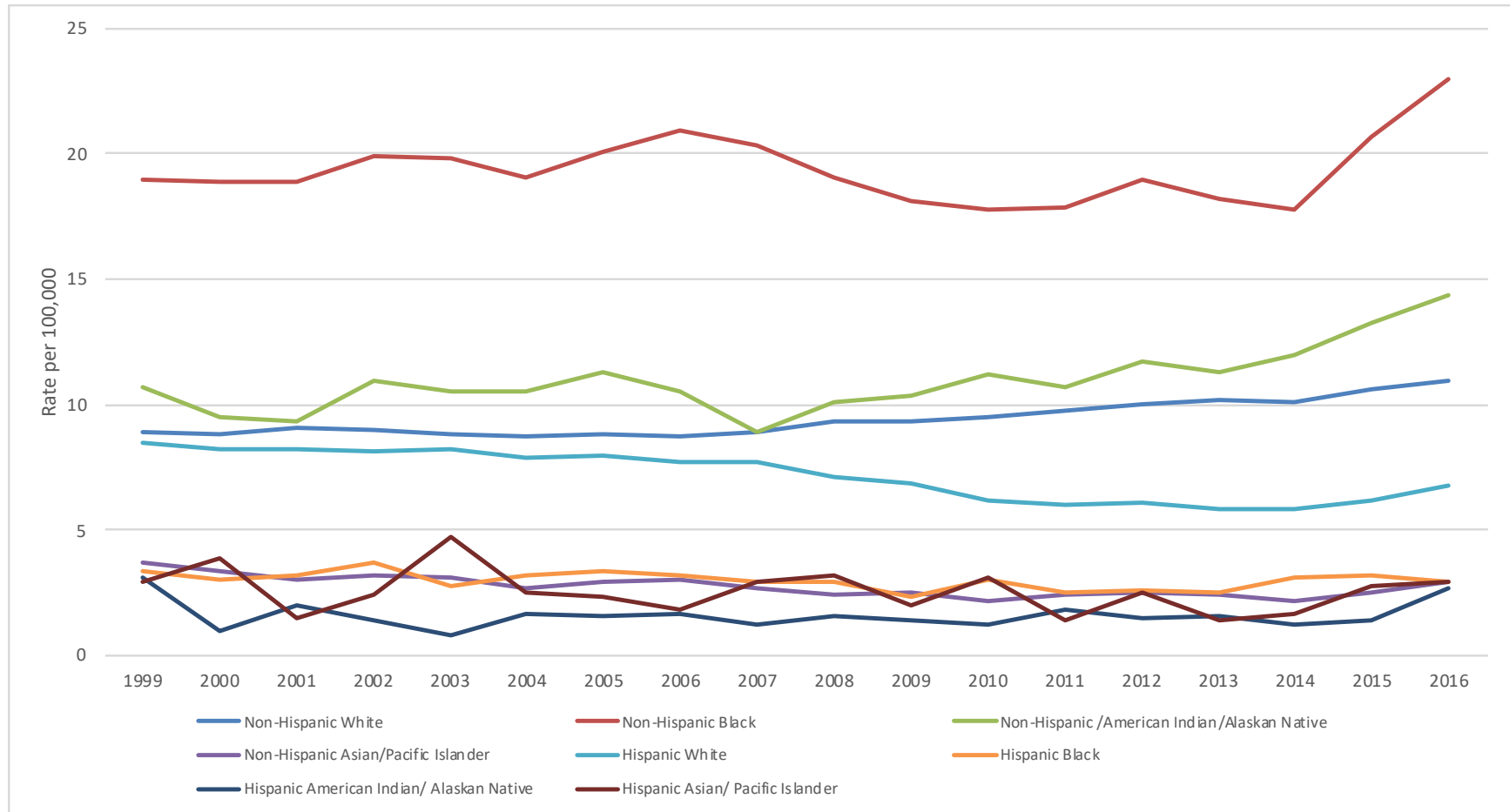

Supplement: S4 Fig — (PDF) [file pone.0225223.s005.pdf]

## Supplementary Appendix

**S5 Fig: National Firearm mortality rates by intent across time, 1999-2016**

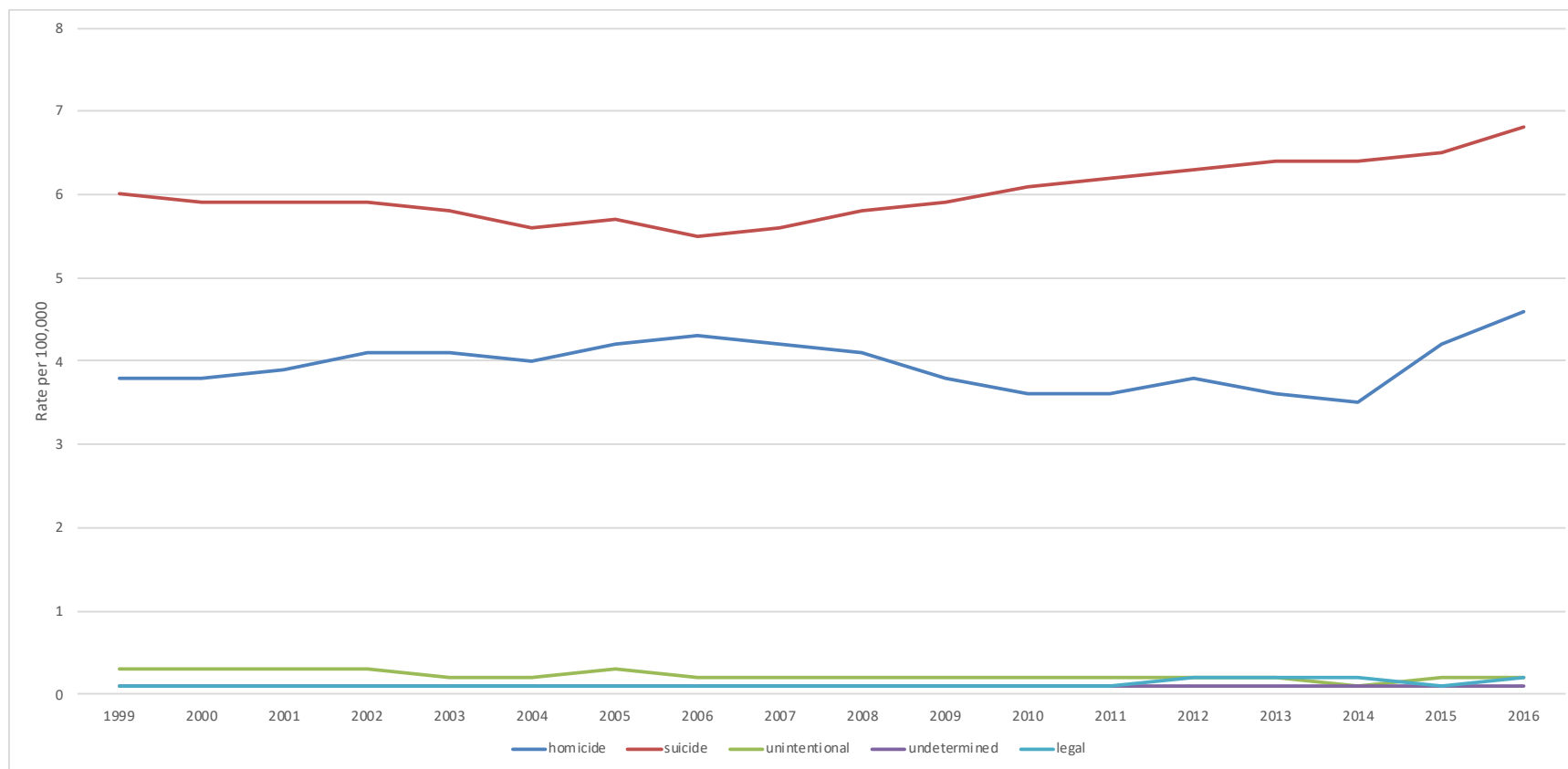

Supplement: S5 Fig — (PDF) [file pone.0225223.s006.pdf]

### Supplementary Appendix

**S6 Fig: National all-cause and Firearm YPLL across time, 1999-2016**

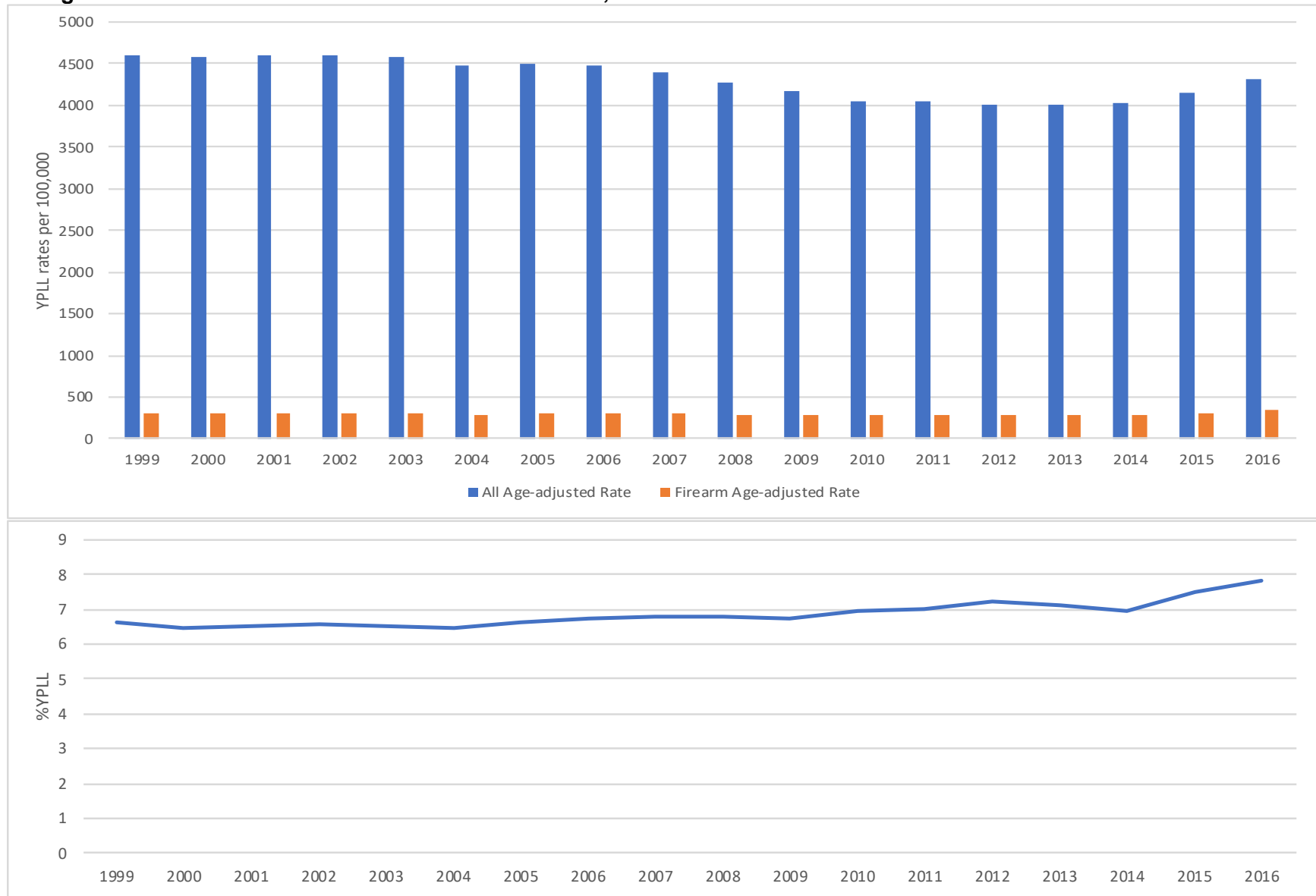

Supplement: S6 Fig — (PDF) [file pone.0225223.s007.pdf]

## Supplementary Appendix

**S7 Fig: National Firearm %YPLL by sex across time, 1999-2016**

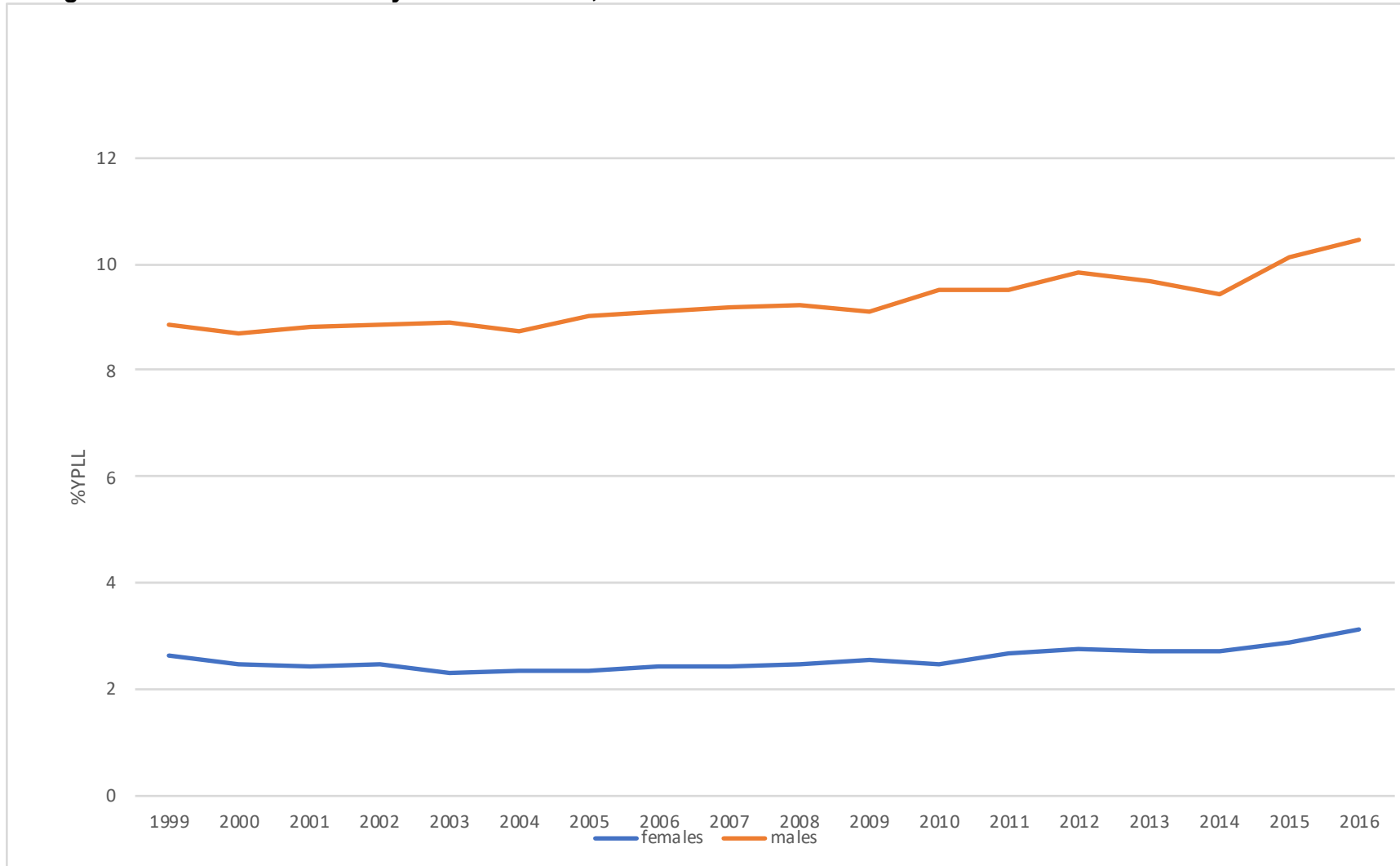

Supplement: S7 Fig — (PDF) [file pone.0225223.s008.pdf]

## Supplementary Appendix

**S8 Fig: National Firearm %YPLL by race/ethnicity across time, 1999-2016**

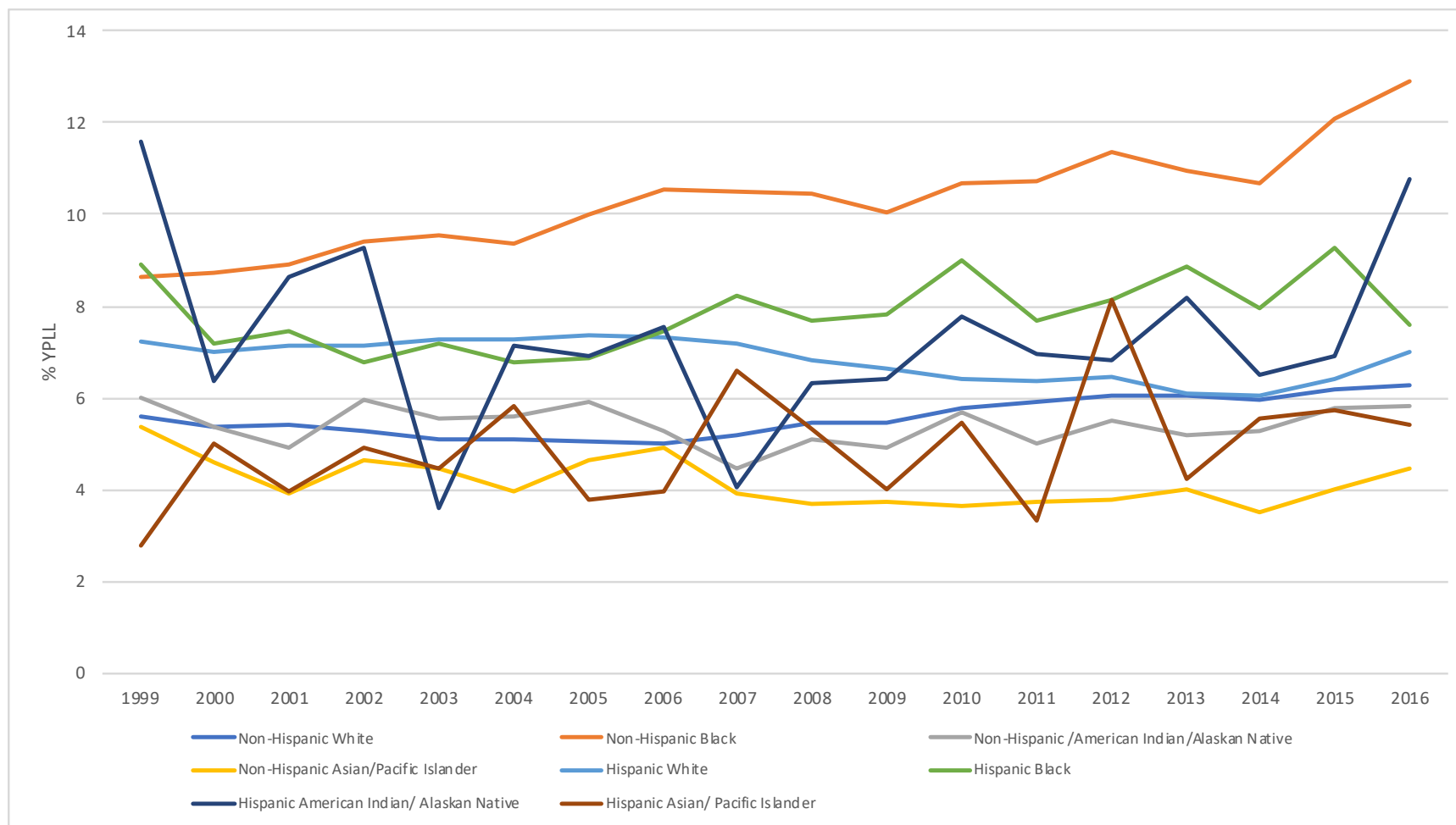

Supplement: S8 Fig — (PDF) [file pone.0225223.s009.pdf]

## Supplementary Appendix

**S9 Fig: National Firearm %YPLL by intent across time, 1999-2016**

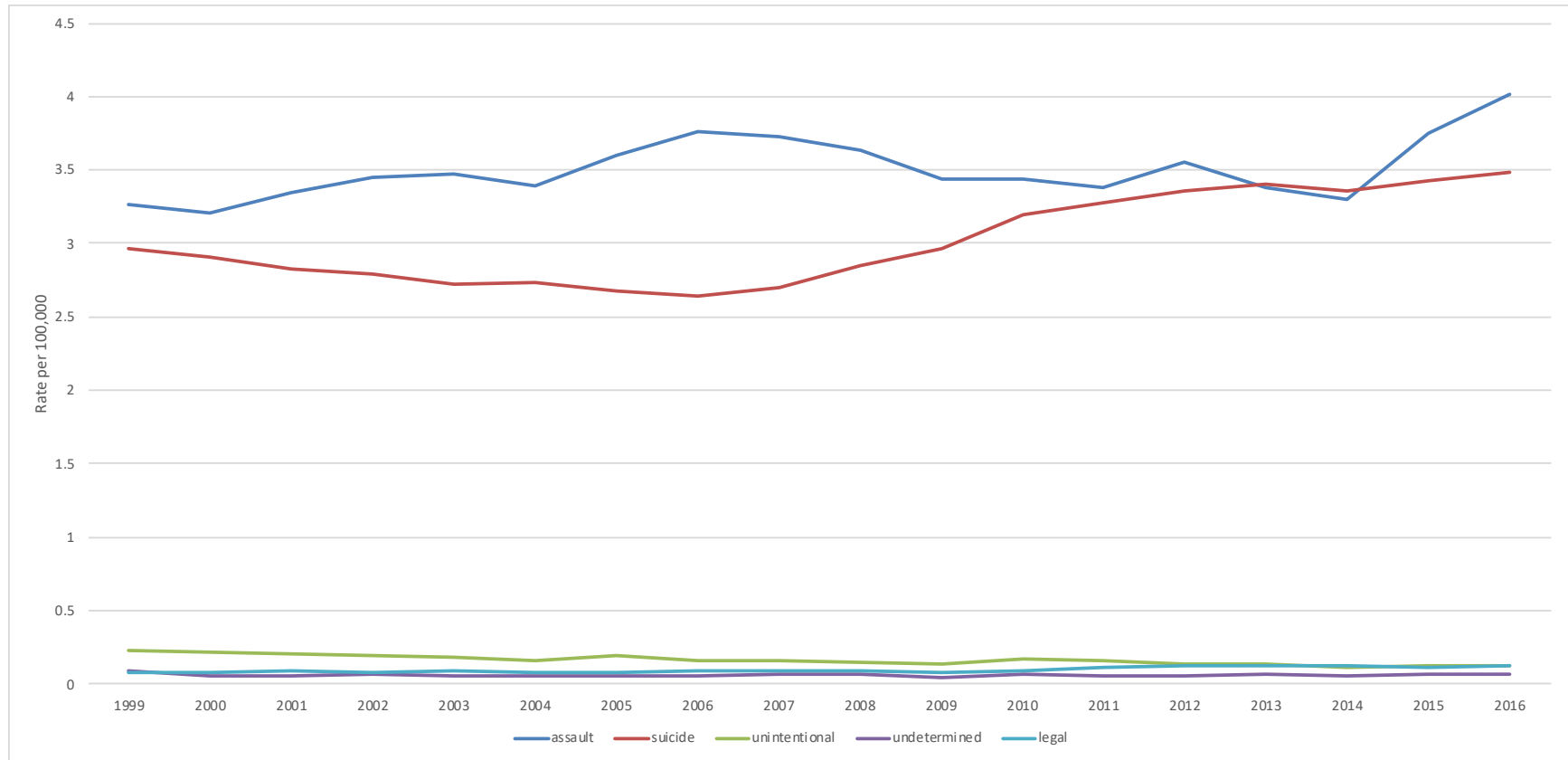

Supplement: S9 Fig — (PDF) [file pone.0225223.s010.pdf]

## Supplementary Appendix

**S14 Fig: State-specific Firearm %YPLL in North-east region across time, 1999-2016**

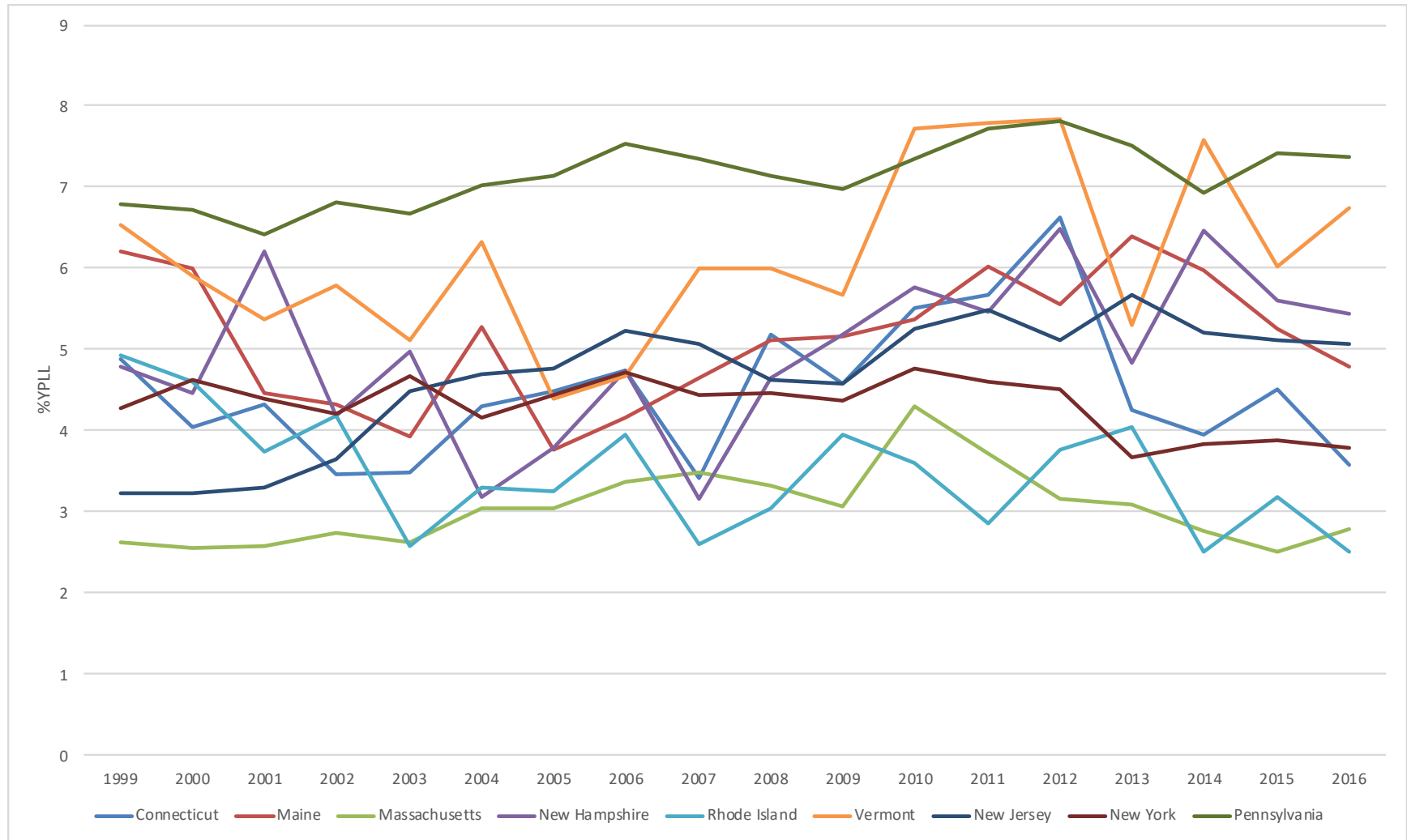

Supplement: S14 Fig — (PDF) [file pone.0225223.s015.pdf]

## Supplementary Appendix

**S15 Fig: State-specific Firearm %YPLL in Midwest region across time, 1999-2016**

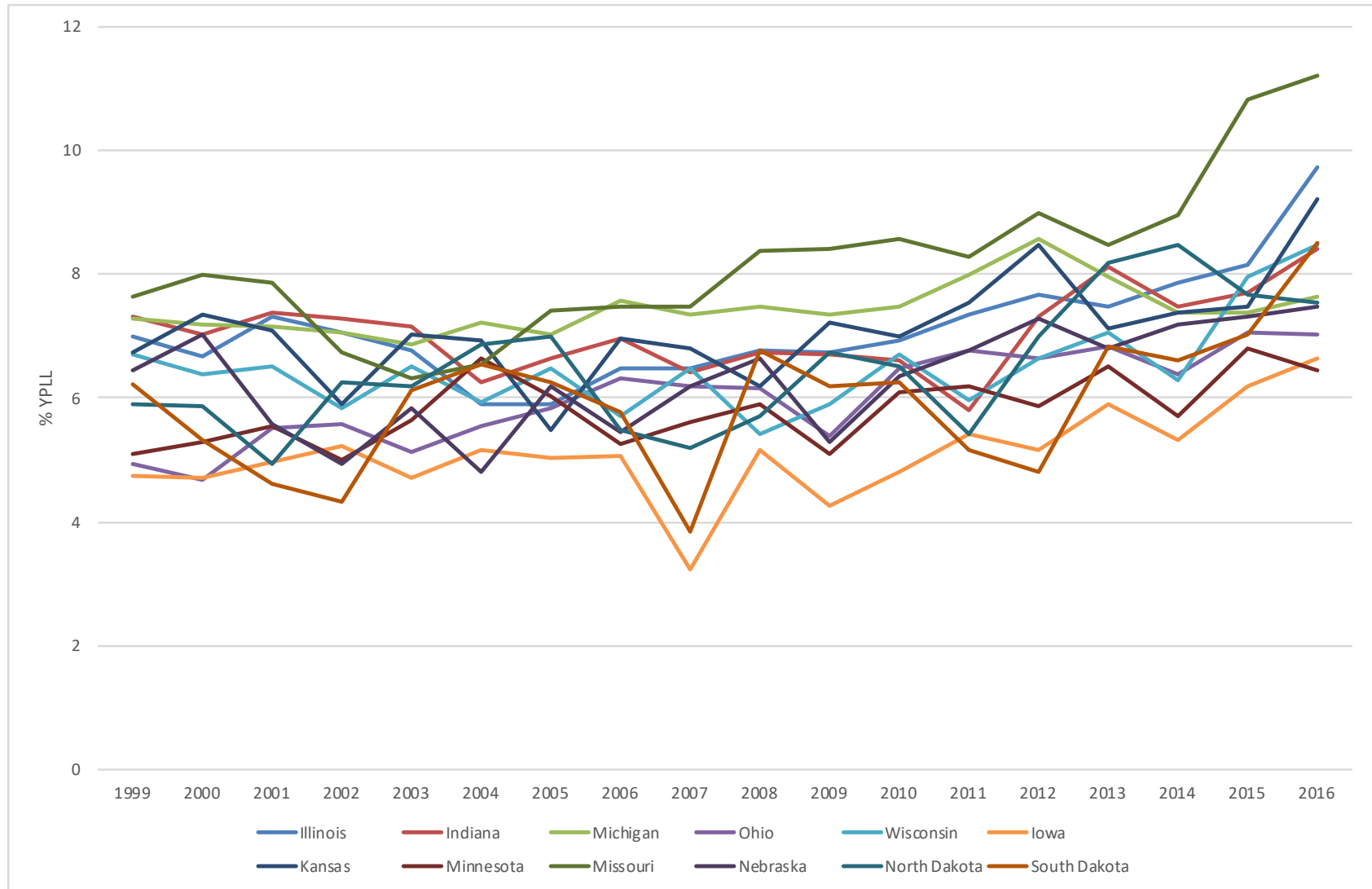

Supplement: S15 Fig — (PDF) [file pone.0225223.s016.pdf]

## Supplementary Appendix

**S16 Fig: State-specific Firearm %YPLL in South region across time, 1999-2016**

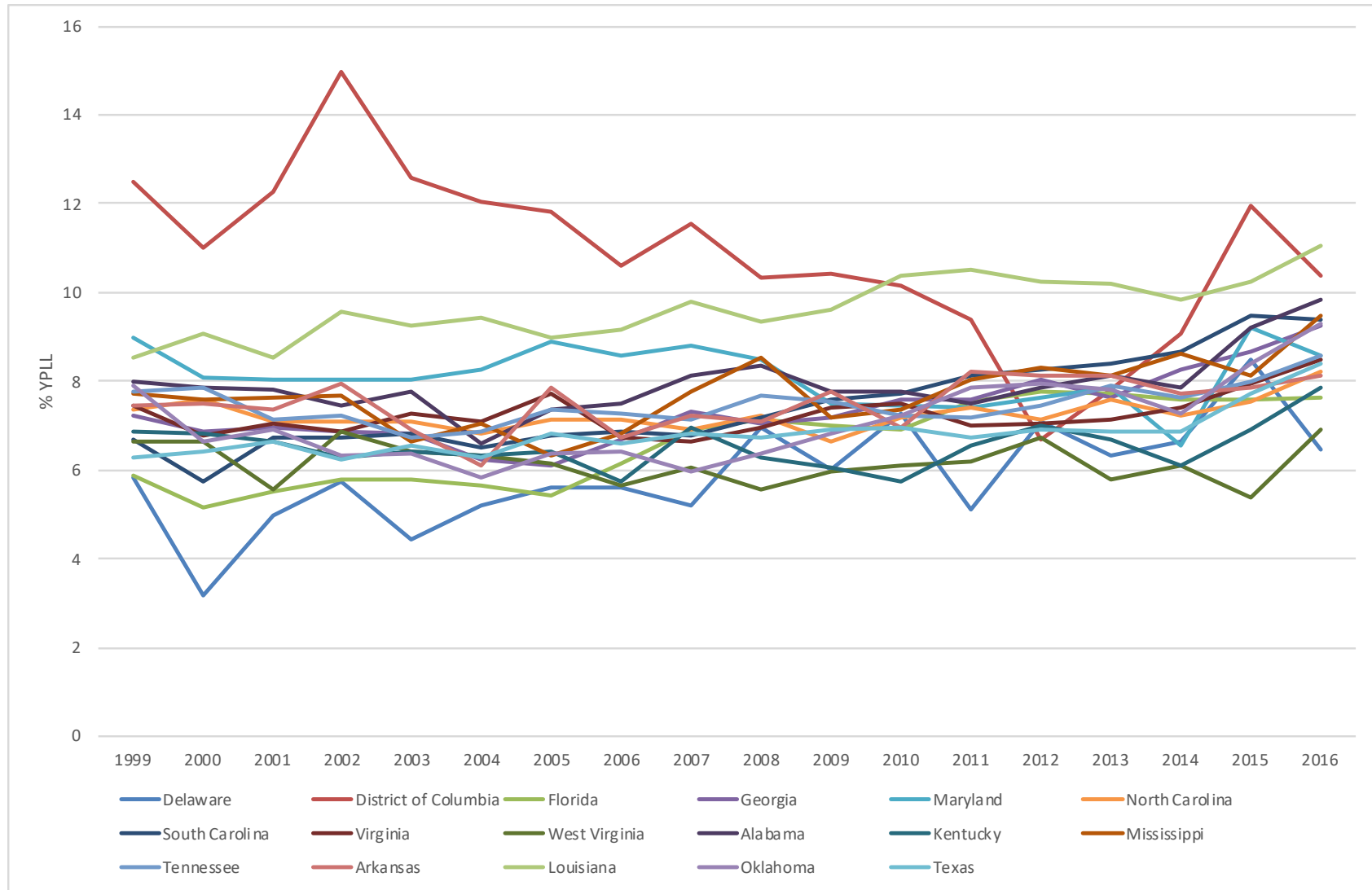

Supplement: S16 Fig — (PDF) [file pone.0225223.s017.pdf]

## Supplementary Appendix

S17 Fig: State-specific Firearm %YPLL in west region across time, 1999-2016

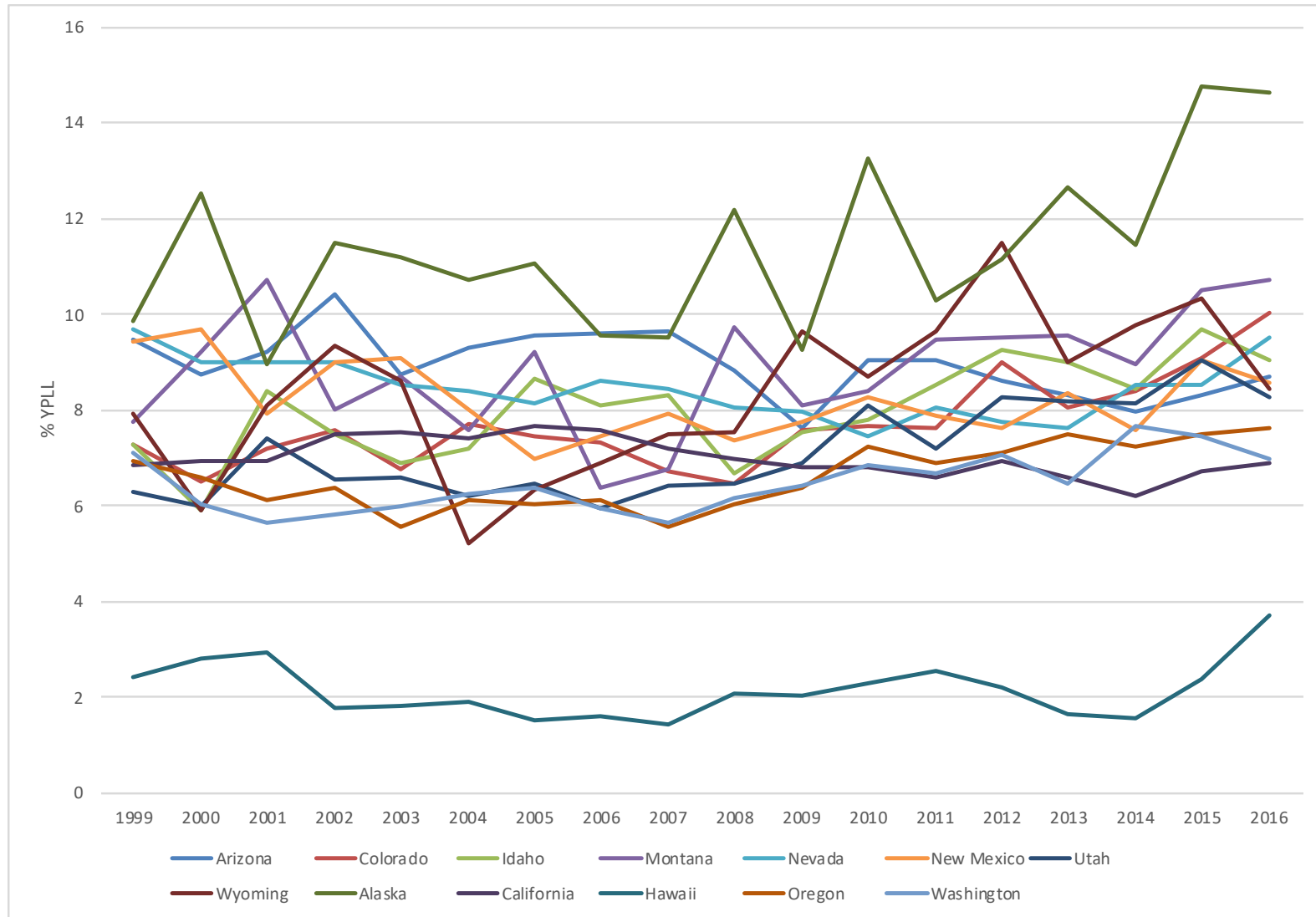

Supplement: S17 Fig — (PDF) [file pone.0225223.s018.pdf]

## Supplementary Appendix

**S18 Fig: Sensitivity analysis using interrupted time series analysis**

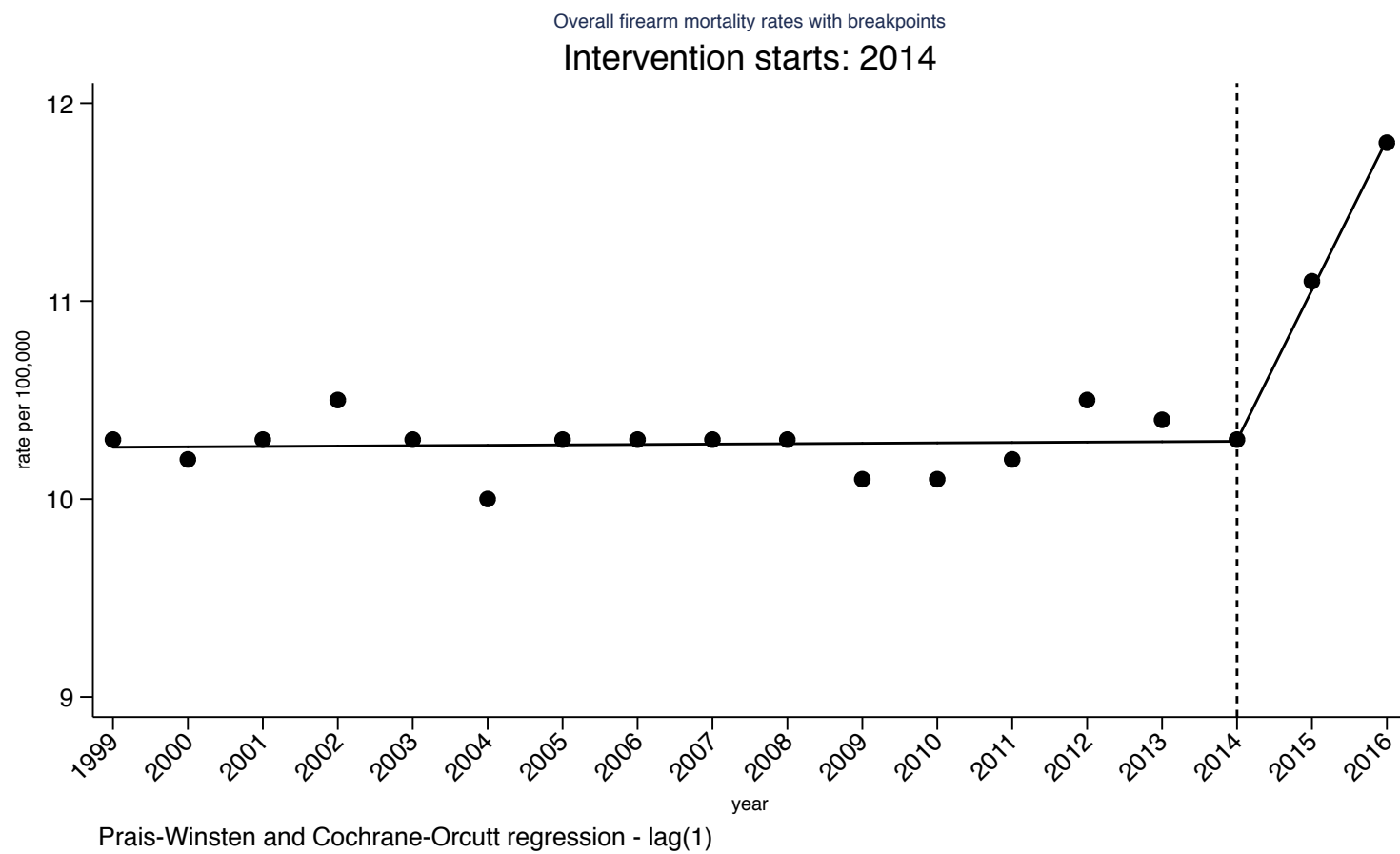

Supplement: S18 Fig — (PDF) [file pone.0225223.s019.pdf]
