# Supplementary material for: Changes in patterns of mortality rates and years of life lost due to firearms in the United States, 1999 to 2016: A joinpoint analysis
Source: PLoS One. 2019 Nov 22;14(11):e0225223. doi: 10.1371/journal.pone.0225223 (PMC6874349; doi:10.1371/journal.pone.0225223)

Supplementary Appendix

S10 Fig: State-specific Firearm mortality rates in North-east region across time, 1999-2016

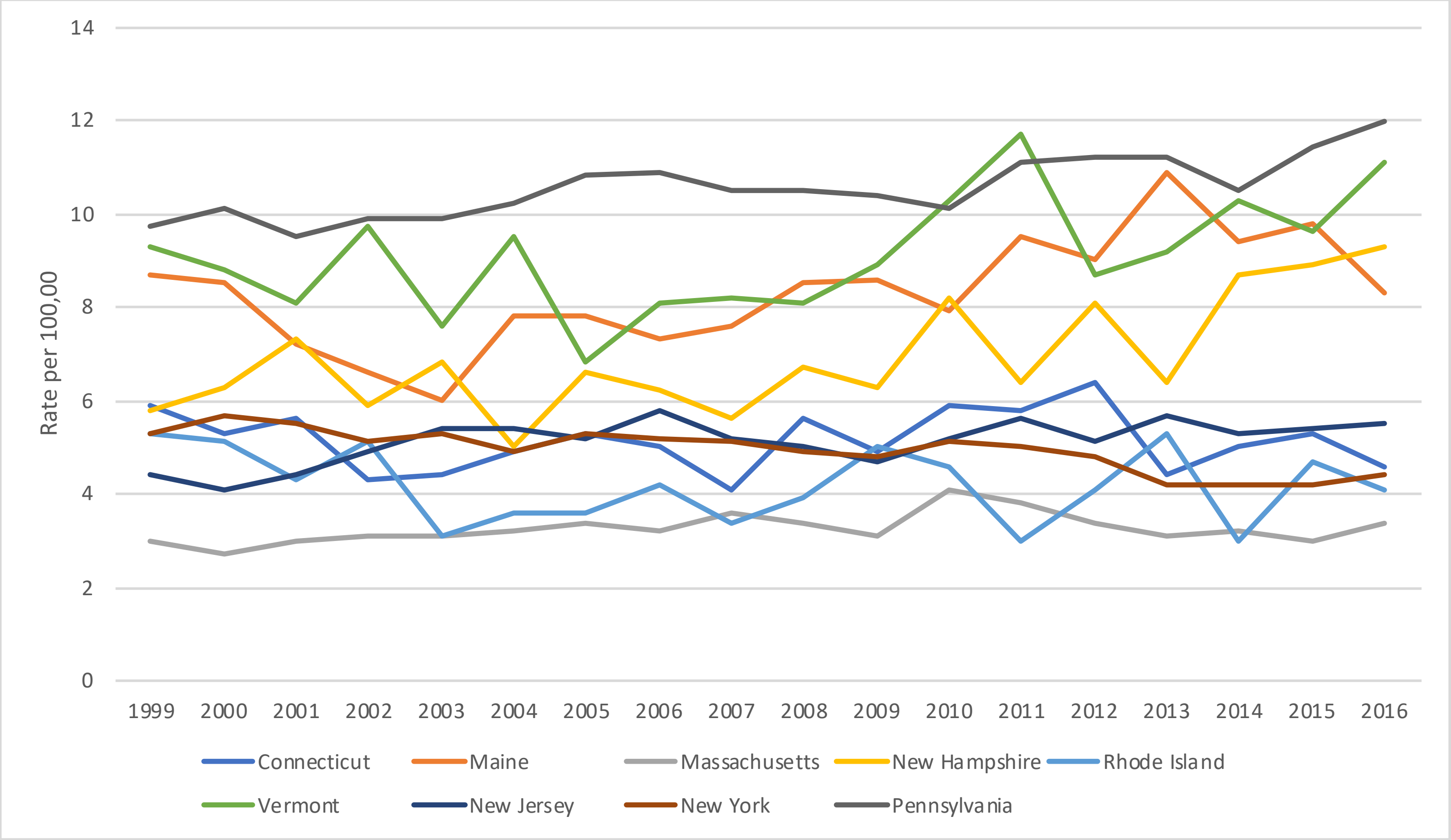

Supplement: S10 Fig — (PDF) [file pone.0225223.s011.pdf]
