# Supplementary material for: Changes in patterns of mortality rates and years of life lost due to firearms in the United States, 1999 to 2016: A joinpoint analysis
Source: PLoS One. 2019 Nov 22;14(11):e0225223. doi: 10.1371/journal.pone.0225223 (PMC6874349; doi:10.1371/journal.pone.0225223)
Supplement: S1 Appendix — (DOCX) [file pone.0225223.s020.docx]

**S1 Appendix: Statistical appendix.**

Piecewise linear regression is a form of regression that allows multiple linear models to be fit to the non-linear data for different ranges of *x*. Breakpoints or inflections are the values of *x* where the slope of the linear function changes. The value of the breakpoint may be known before the analysis, but typically it is unknown and must be estimated before the analysis. Although the regression function at the breakpoint may be discontinuous, a model can have a function that is continuous at all points, including the breakpoints in piecewise regression. However, joinpoint regression is different from temporal trends models like piecewise regression, because it has the constraint of continuity at the change-points and the choice of the number of joinpoints and their locations is estimated within the model.[1-4] A distinguishing characteristic of this model is that the minimum and the maximum number of joinpoints allowed is arbitrarily set before the analysis while the final number of joinpoint(s) is not fixed a priori, as in a classical piecewise regression model, but established based on a statistical criterion. The analysis was implemented in the Joinpoint Regression Program (V4.1.1, National Cancer Institute).[5, 6]

**References**

1. Ma J, Ward EM, Siegel RL, Jemal A. Temporal Trends in Mortality in the United States, 1969-2013. JAMA. 2015;314(16):1731-9. Epub 2015/10/28. doi: 10.1001/jama.2015.12319. PubMed PMID: 26505597.

2. Feder PI. On Asymptotic Distribution Theory in Segmented Regression Problems: Identified Case. Annals of Statistics. 1975;3:49-83.

3. Lerman PM. Fitting Segmented Regression Models by Grid Search.

4. Hudson DJ. Fitting Segmented Curves Whose Join Points Have to be Estimated.

5. Joinpoint Regression Program [computer software]. version 4.5.0.1. Bethesda, MD; Statistical Research and Applications Branch, National Cancer Institute; June 2017.

6. Joinpoint Regression Program; Statistical Methodology and Applications Branch SRP, National Cancer Institute. Version 4.5.0.1 ed2017.
